# Constraining the intensive absorption properties of ambient organic aerosol particles based on pan-European observations

**DOI:** 10.1038/s41612-026-01405-9

**Published:** 2026-04-10

**Authors:** Jordi Rovira, Jesús Yus-Díez, Gang I. Chen, Griša Močnik, Martin Gysel-Beer, Wenche Aas, Minna Aurela, John Backman, Sujai Banerji, Benjamin T. Brem, Anna Canals-Angerri, Benjamin Chazeau, Kaspar R. Daellenbach, Joel F. de Brito, Evangelia Diapouli, Konstantinos Eleftheriadis, Mikael Ehn, Olivier Favez, Harald Flentje, Maria I. Gini, Konstantinos Granakis, Asta Gregorič, Roy Harrison, Liine Heikkinen, Christoph Hueglin, Antti Hyvärinen, Matic Ivančič, Hannes Keernik, Eleni Liakakou, Chunshui Lin, Radek Lhotka, Krista Luoma, Marek Maasikmets, Hanna E. Manninen, Manousos Ioannis Manousakas, Nicolas Marchand, Saliou Mbengue, Nikos Mihalopoulos, María Cruz Minguillón, Doina Nicolae, Jarkko V. Niemi, Jurgita Ovadnevaite, Noemí Pérez, Jean-Eudes Petit, Stephen M. Platt, Petra Pokorná, André S. H. Prévôt, Véronique Riffault, Martin Rigler, Matteo Rinaldi, Jaroslav Schwarz, Iasonas Stavroulas, Erik Teinemaa, Kimmo Teinilä, Hilkka Timonen, Anna Tobler, Jeni Vasilescu, Marta Via, Petr Vodička, Stergios Vratolis, Karl Espen Yttri, Naděžda Zíková, Olga Zografou, Andrés Alastuey, Tuukka Petäjä, Xavier Querol, Marco Pandolfi

**Affiliations:** 1 Institute of Environmental Assessment and Water Research (IDAEA-CSIC), Barcelona, Spain; 2 Department of Applied Physics-Meteorology, Universitat de Barcelona, Barcelona, Spain; 3 Center for Atmospheric Research, University of Nova Gorica, Ajdovščina, Slovenia; 4 Environmental Research Group, MRC Centre for Environment and Health, Imperial College London, London, UK; 5 Haze Instruments d.o.o., Ljubljana, Slovenia; 6 Department of Environmental Sciences, Jozef Stefan Institute, Ljubljana, Slovenia; 7 PSI Center for Energy and Environmental Sciences, Villigen PSI, Switzerland; 8 NILU, Kjeller, Norway; 9 Atmospheric Composition Research, Finnish Meteorological Institute, Helsinki, Finland; 10 Institute for Atmospheric and Earth System Research/Physics (INAR), Faculty of Science, University of Helsinki, Helsinki, Finland; 11 Aix Marseille Univ., CNRS LCE, Marseille, France; 12 IMT Nord Europe, Centre for Energy and Environment, Douai, France; 13 ENRACT, Institute of Nuclear and Radiological Science & Technology, Energy & Safety, National Centre for Scientific Research “Demokritos”, Athens, Greece; 14 Institut National de l’Environnement Industriel et des Risques (INERIS), Verneuil-en-Halatte, France; 15 German Meteorological Service (DWD), Observatory Hohenpeissenberg, Hohenpeissenberg, Germany; 16 Aerosol d.o.o., Ljubljana, Slovenia; 17 School of Geography, Earth & Environmental Sciences, University of Birmingham, Edgbaston, Birmingham, UK; 18 Laboratory for Air Pollution and Environmental Technology, Swiss Federal Laboratories for Materials Science and Technology (Empa), Duebendorf, Switzerland; 19 Estonian Environmental Research Centre, Air Quality Management Department, Tallinn, Estonia; 20 Institute of Physics, University of Tartu, Tartu, Estonia; 21 Institute for Environmental Research & Sustainable Development, National Observatory of Athens, Athens, Greece; 22 School of Natural Sciences, Physics, Centre for Climate and Air Pollution Studies, Ryan Institute, University of Galway, University Road, Galway, Ireland; 23 Institute of Chemical Process Fundamentals of the Czech Academy of Sciences, Prague, Czechia; 24 Helsinki Region Environmental Services Authority (HSY), Helsinki, Finland; 25 Global Change Research Institute, Czech Academy of Sciences, Brno, Czechia; 26 National Institute of Research and Development for Optoelectronics INOE, Magurele, Romania; 27 Laboratoire des Sciences du Climat et de l’Environnement, CEA/Orme des Merisiers, Gif-sur-Yvette, France; 28 Institute of Atmospheric Sciences and Climate (ISAC), National Research Council (CNR), Bologna, Italy; 29 Aerosol Physics Laboratory, Faculty of Engineering and Natural Sciences, Tampere University, Tampere University, Tampere, Finland; 30 Datalystica Ltd., Villigen, Switzerland

**Keywords:** Climate sciences, Environmental sciences

## Abstract

Organic aerosol particles (OA) can absorb solar radiation with varying efficiencies depending on their chemical composition and physical properties. This light-absorbing fraction of OA, commonly referred to as brown carbon (BrC), is difficult to accurately represent in climate models due to the inherent diversity of its optical properties. This variability arises from differences in emission sources and atmospheric processing, as well as from variations in experimental design and the analytical methods used to quantify BrC absorption. As a result, the climate effect of BrC remains uncertain. Here, we studied the light absorption properties of surface ambient OA using measurements from 17 sites across Europe. Combining multi-wavelength absorption measurements from filter-based photometers with OA mass concentrations and source apportionment derived from ACSM/AMS data, we derive empirical estimates of the OA mass absorption cross section (MAC_OA_), its wavelength dependence (AAE_OA_), the OA density (⍴_OA_), and the MAC associated with different primary and secondary OA sources. We further develop parameterizations that relate MAC_OA_, AAE_OA_ and ⍴_OA_ to the ambient black carbon-to-organic aerosol ratio (eBC/OA) and propose a corresponding parameterization for the imaginary refractive index (k_OA_). Given the widespread availability of eBC and OA measurements in global monitoring networks, the framework presented here provides a practical approach for estimating the absorptive properties of surface OA particles under real-world conditions.

## Introduction

Brown carbon (BrC) defines a class of organic aerosols (OA) that efficiently absorb light at ultraviolet (UV) wavelengths and less significantly in the visible part of the spectrum^1–6^. BrC is a complex collection of light-absorbing organic molecules that can be soluble in solvents (such as water, methanol, and acetone) or insoluble. A specific type of insoluble and more refractory BrC, often referred to as dark-BrC or BrC tarballs, is more strongly light-absorbing than soluble BrC and has been observed in near-source environments, such as plumes from open biomass burning, wildfires, or ship engine emissions^7–10^. Given that OA constitutes a large fraction, ranging from 20% to 90%, of atmospheric aerosols^11^, its radiative forcing at the top of the atmosphere is considerable especially in regions with strong BrC emissions. Globally, biomass burning (BB) and biofuel (BF) have been recognized as major sources of both primary and secondary BrC^12–21^ and many climate models consider BB and BF as the primary—if not sole—sources of BrC^22,23^. However, recent field and laboratory studies have shown that other fossil sources, such as vehicular emissions, cooking, and coal combustion, can also produce light-absorbing BrC^18,24–27^. This complexity makes it challenging to accurately estimate the absorption properties of OA in climate models. In addition to combustion sources, recent evidence indicates that secondary organic aerosols (SOA) formed in the atmosphere through multi-phase reactions of various biogenic and anthropogenic precursors (i.e., volatile organic compounds, VOCs) in the presence of high NOx and NH₃ concentrations can also contribute to light-absorbing organic aerosols^28–30^. However, light-absorbing SOA generally exhibits relatively short atmospheric lifetimes, as these particles can be rapidly oxidized, either by photobleaching or oxidative aging, in the atmosphere^29,30^. Therefore, SOA contribution to absorption is expected to be most relevant shortly after formation and on local to regional scales, representing a limitation when assessing its importance in aged air masses and long-range transport. Despite growing evidence of BrC’s contribution to radiative forcing, the incomplete characterization of its light-absorbing properties continues to hinder accurate estimates of its climate effects, with only limited progress in recent years^31–35^.

The key parameters used in climate models to quantify the light-absorbing properties of OA particles are the mass absorption cross section (MAC_OA_), the mass absorption efficiency (MAE_OA_) and the imaginary refractive index (*k*_*OA*_)^23^. As described below, MAC_OA_, MAE_OA_, and *k*_OA_ are closely related but describe absorption from different perspectives and units. Another important physical property of OA is its density (⍴_OA_), which is closely linked to chemical composition and provides insights into particle emissions and aging processes^33,36–38^. The Ångström exponents of MAC_OA_ and *k*_OA_ (commonly referred to in the literature as AAE_OA_ and w, respectively) are also widely used to describe the wavelength dependence of these intensive optical properties^5,39^. Several methods have been used to study the light-absorbing properties of BrC. One offline approach, involving detailed laboratory analysis, directly measures the absorbance spectra of organics extracted in water or organic solvents (e.g., methanol) using UV–Vis spectroscopy coupled with a long-path detection cell^40–46^. The ratio between the measured absorbance and the mass of the dissolved OA fraction provides the mass absorption efficiency (MAE_OA_) that can be directly related to the *k*_OA_ assuming suitable organic material density^44,47^. The main drawback of this approach is that the measured absorption properties of soluble organics depend on the solvent used, and not all organics can be extracted, leaving insoluble substances unmeasured and limiting direct comparisons with in situ ambient OA absorption. Furthermore, an often-overlooked issue is that the *k*_OA_ obtained using this method may differ from the true *k* of the absorbing material in its pure form unless the solvent and solute have identical refractive indices^43^. A widely used online approach to study the absorption properties of ambient BrC employs multi-wavelength absorption measurements—typically using filter-based photometers—combined with determination of the OA mass. This method (referred to as attribution method) takes advantage of the different spectral dependencies of absorption by black carbon (eBC) and BrC. Using this method, the contribution of BrC to total absorption is estimated by subtracting to the total absorption the absorption attributable to eBC. Using this approach, the ratio of the estimated BrC absorption to the OA mass yields the mass absorption cross section (MAC_OA_) of ambient OA particles. This method is particularly appealing for its simplicity, as it requires only multi-wavelength absorption measurements and OA mass data. A key limitation of this approach is that filter-based photometers, such as the AE33 aethalometer, are subject to instrument-specific uncertainties associated with particle collection on a filter substrate. Although the dual-spot technology implemented in the AE33 substantially mitigates filter loading effects, the measured light attenuation still relies on empirical correction factors accounting for multiple scattering and filter–particle interactions. These correction factors may vary with aerosol composition and size distribution^48,49^. In addition, accurate application of this method requires an appropriate value for the Ångström exponent of eBC (AAE_BC_)^6,18,20,50^ which depends on eBC properties such as particle size, morphology, and mixing state. Finally, another online approach for estimating BrC absorption properties is optical closure, which integrates real-time measurements of optical properties (absorption and scattering), particle size distributions, eBC and OA mass concentrations, and the chemical composition of non-refractory components to retrieve MAE_OA_ and *k*_OA_. This method typically relies on literature values for the eBC refractive index and involves assumptions regarding the shape and mixing state of eBC and OA particles that can introduce uncertainties in simulated eBC and BrC absorption^29,39^. Furthermore, the applicability of this approach is limited by the need for a comprehensive set of measurements, which are not available at all monitoring stations.

In the literature, reported values of MAC_OA_, MAE_OA_, and *k*_OA_ exhibit high variability, depending on the methodology used, the type of experiment (e.g., chamber versus ambient measurements), the OA mass to which the measured absorption is attributed (e.g., mass of absorbing molecules, total OA, or specific OA sources), and the physico-chemical properties of the BrC particles, which are influenced by the source (e.g., fuel type), combustion conditions, and atmospheric processing^5,44^. Some works have reported parameterizations that can be used to calculate the *k*_OA_ of OA particles from the eBC/OA ratio^39,45,51–53^. However, these parameterizations are primarily based on data from controlled BB/BF combustion experiments or field measurements near strong BB/BF emission sources, which may not fully reflect ambient conditions^35^. In fact, ambient or remote-sensing data were excluded from these previous studies, as they can be influenced by emissions from multiple BrC sources and by the presence of SOA (e.g., ref. ^51^ and references herein). Additionally, in controlled experiments, the eBC/OA ratio has been used primarily as an indicator of burning conditions rather than the type of fuel burned^23,54^. Consequently, these parameterizations are difficult to apply because in emissions inventories the eBC/OA ratio primarily reflects differences in fuel types and does not include information on burning conditions^23^. To characterize the absorption properties of atmospheric OA under ambient conditions, we present and discuss empirically derived MAC_OA_ values based on measurements from 17 sites across Europe, where absorption was measured using filter-based photometers (aethalometer, AE33) and OA mass was determined with ACSM/AMS instruments. The OA mass concentration measurements and OA source apportionment at these sites were presented in the research^55^ and used here in combination with absorption measurements. We present parameterizations that describe the relationships between MAC_OA_, MAC_OA_ Ångström exponent (AAE_OA_) and *⍴*_OA_ of OA particles and the ambient measurements of the eBC/OA ratio. Since similar parameterizations in the literature exist primarily for *k*, we also propose, as detailed in the Methods section, a parameterization for *k*_OA_ and compare it with previous studies. The utility of this work lies in the widespread availability of ambient eBC and OA measurements in global databases such as EBAS (www.ebas.no), which are also routinely simulated by models^35,56^ and can be used within the framework presented here to estimate the absorption properties of ambient OA particles.

## Results

Here, we present and discuss the absorption properties derived for bulk OA and for individual OA sources across the 17 measurement sites, as well as their parameterization as a function of log(eBC/OA).

### MAC_OA_ and AAE_OA_ of OA sources

**Fig. 1:**
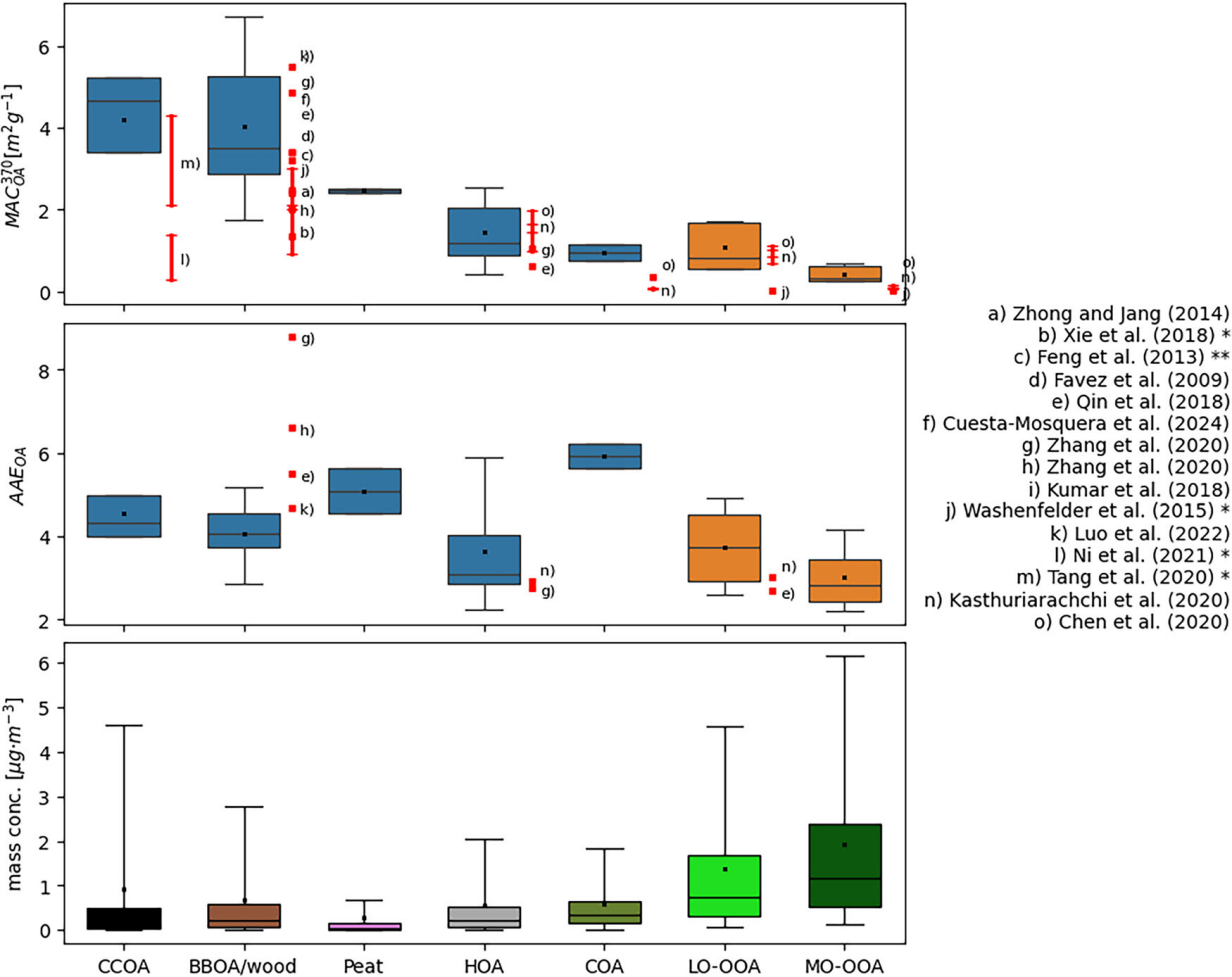
Variability of BrC optical properties (MAC₃₇₀ and AAE_OA_) with OA source contributions. Box plots (5th, 25th, 50th, 75th, 95th percentiles) of MAC_OA_ at 370 nm and AAE_OA_ between 370 and 590 nm of OA sources obtained from MLR analysis. The bottom panel reports the average source contribution to OA as reported in the research^55^. Horizontal black lines and dots in each box-whiskers plot represent the median and mean values, respectively. Blue and orange colors are used for POA and SOA, respectively. Red vertical lines and red dots represent the values at 370 nm reported in literature. * 365 nm; **350 nm. g and h are the MAC_OA_ values of MO-OOA and LO-OOA.

Figure 1 shows the MAC_OA_ at 370 nm and the AAE_OA_ (370–590 nm) obtained for the main OA sources identified at the various measurement sites, and compares these results with values reported in the literature. Figure 1 also reports the average OA source contributions from all measurement sites included here^55^. The MAC_OA_(*λ*) and AAE_OA_ values of the sources identified at every single site are provided in Table S2. As commented in the “Method” section, the MAC values represent the average MAC across all possible absorptive conditions of each OA source.

Overall, Fig. 1 shows higher MAC for POA sources compared to SOA sources, consistent with literature findings^16,18,57^, whereas a clear trend for AAE was not observed. Figure 1 also indicates that SOA (and in particular MO-OOA), despite its relatively low MAC, contributes on average more to OA than POA sources and may therefore have a non-negligible impact on climate. The main OA sources reported for Europe in Chen^55^, and studied optically in this work, were BBOA/Wood (biomass burning; identified at 15 sites out of 17 sites included here), CCOA (coal combustion; 3 sites out of 17), Peat combustion (2/17), COA (cooking; 5/17), HOA (vehicle exhaust; 16/17), less oxidized (16/17) and more oxidized (16/17) oxygenated OA (LO-OOA and MO-OOA, respectively). For BBOA, CCOA and Peat combustion, the MLR analysis yielded statistically significant higher MAC values (see Table S2) across all sites where these sources were present, confirming that they are efficient sources of BrC particles. CCOA, followed by BBOA/wood, showed the highest MAC compared to the other POA sources (4.66 ± 1.87 m^2^g^−1^ and 3.52 ± 1.72, respectively). This was likely related to the high content in both BBOA and CCOA of condensed aromatic structures (PAHs, oxygenated PAHs, polyphenols), which are strong chromophores absorbing UV and visible light^58,59^. BBOA MAC values at 370 nm ranged from around 1.7 m^2^ g^−1^ at BIR and DEM to more than 6 m^2^ g^−1^ at ZUR and KOS (cf. Table S2). Diapouli^60^ reported, using data collected at DEM during 2016–2017, that this site is more frequently affected by transport of aged wildfire smoke plumes, rather than by local biomass burning for residential heating. Thus, at DEM the low MAC of BBOA reported here could be related to the loss of BrC during transport through photobleaching processes and/or internal mixing with eBC. Conversely, the high BBOA MAC observed at ZUR could be due to the direct emissions from close sources as restaurants and residential areas in the immediate vicinity of the measurement site^61^. The standard deviation of BBOA MAC was around 50% reflecting the variability of the absorption efficiency of BBOA particles related to the fuel burned, burning conditions, distance of the measurement site from the source, aging and transformation processes during transport. The observed variability in BBOA MAC was consistent with the wide range of values reported in the literature (cf. Fig. 1). Reported MAC values span from 1–2 m^2^ g^−1^ for various biofuel types in prescribed and laboratory burns^62^, to 2–3 m^2^ g^−1^ depending on aging time and the extent of photobleaching in chamber experiments^63^, and up to 3–5 m^2^ g^−1^ in ambient measurements of agricultural waste burning, biomass burning, and residential wood-burning emissions^18,20,30,34,64^. For CCOA, the MAC showed a rather high standard deviation of around 40%. Estimations of the MAC of coal combustion OA are scarce in the literature and were obtained mostly from smog chamber experiments^27,65^.

Among primary sources of OA, HOA and COA showed lower MAC (1.17 ± 1.07 and 0.95 ± 0.60 m^2^ g^−1^, respectively). It should be noted that the MAC for HOA was statistically significant at 13 of the 16 sites, while the MAC for COA was significant at two of the five sites (cf. Table S2). Thus, at some sites HOA and COA were not detected as sources of BrC, and the MLR results yielded non-statistical significant near-zero MAC values. This may be attributed to several factors, including differences in vehicle fleets for HOA and variations in ingredients (e.g., meats, fish, vegetables), cooking methods (boiling, roasting, frying), and stove types (gas, electric, wood, charcoal) for COA. As reported in Fig. 1, the HOA and COA MAC values reported here were in the range of variability of MAC values reported in literature for HOA and were somewhat higher for COA^18,20,21,26^. A MAC was also calculated for CSOA (cigarette smoke) detected only at ZUR with a value of 0.75 m^2^g^−1^ at 370 nm (cf. Table S2). Peat combustion was only identified as a source of BrC particles at DUB and CASP^55^, where it showed consistent MAC values of around 2.4 and 2.6 m^2^ g^−1^ at 370 nm, respectively. The MO-OOA median MAC (0.32 ± 0.21 m^2^ g^−1^) was lower compared to the MAC of LO-OOA (0.80 ± 0.67 m^2^ g^−1^), likely reflecting the effect of aging and photobleaching of the more oxidized OOA. Moreover, the MAC of LO-OOA and MO-OOA were nonzero at 7 and 9 of the 16 sites where these sources were detected, respectively. This reflects the high variability in the absorption properties of ambient secondary organic aerosols, which is influenced by the nature of the OOA precursors and the availability of browning agents such as NH₃ and/or NOx during OOA formation^28^. The OOA MAC values reported here were comparable to those previously published^26^.

The Ångström exponent of the calculated median MAC values (AAE; Fig. 1) showed less source-to-source variability compared to the MAC and decreased in the following order: 5.91 ± 0.31 (COA), 5.09 ± 1.02 (Peat), 4.63 ± 0.75 (BBOA), 4.47 ± 0.89 (CCOA), 4.23 ± 1.25 (HOA), 4.02 ± 1.03 (LO-OOA), and 3.35 ± 0.70 (MO-OOA). Note that the standard deviation for the AAE was rather low for all sources and ranged between 5% (for COA) and 30% (for HOA). For CSOA the AAE was 3.65 (cf. Table S2). Similar to MAC, AAE values reported in the literature exhibit substantial variability, reflecting differences in factors such as aging times and oxidation conditions of OA particles. AAE values of BBOA and HOA (370–660 nm) between 3.3 and 5.5 and 2.8 and 3.5, respectively, were reported by previous studies^18,20,21,26^. Larger AAE values between around 5 up to more than 8 (240/300–550 nm) were reported for different biofuels by previous studies^62,66^. As reported above, here we found that the AAE of OOA particles was on average lower compared to the AAE of POA, with AAE of LO-OOA and MO-OOA within 3.0–5.8 and 2.7–5.1, respectively. It should be noted that higher AAE values for OOA than for POA have been reported in literature mostly from chamber experiments and were associated with a sharper decrease of OOA MAC with the wavelength^5^. However, also the AAE of OOA reported in the literature varied considerably depending on the OOA precursors and formation. For example, the research^5^ reported AAE from chamber experiments of around 5–9 for SOA from aromatic VOCs and lower values around 3-4 for SOA from biomass burning. Higher AAE at 370 nm for ambient LO-BBOA and MO-BBOA (6.6 and 8.8, respectively) were reported by Zhang^20^, whereas lower LO-OOA AAE around 2.7–3.0 were reported by previous studies^18,26^.

### MAC_OA_, AAE_OA_, *⍴*_OA_, and *k*_*OA*_ of bulk OA

**Fig. 2:**
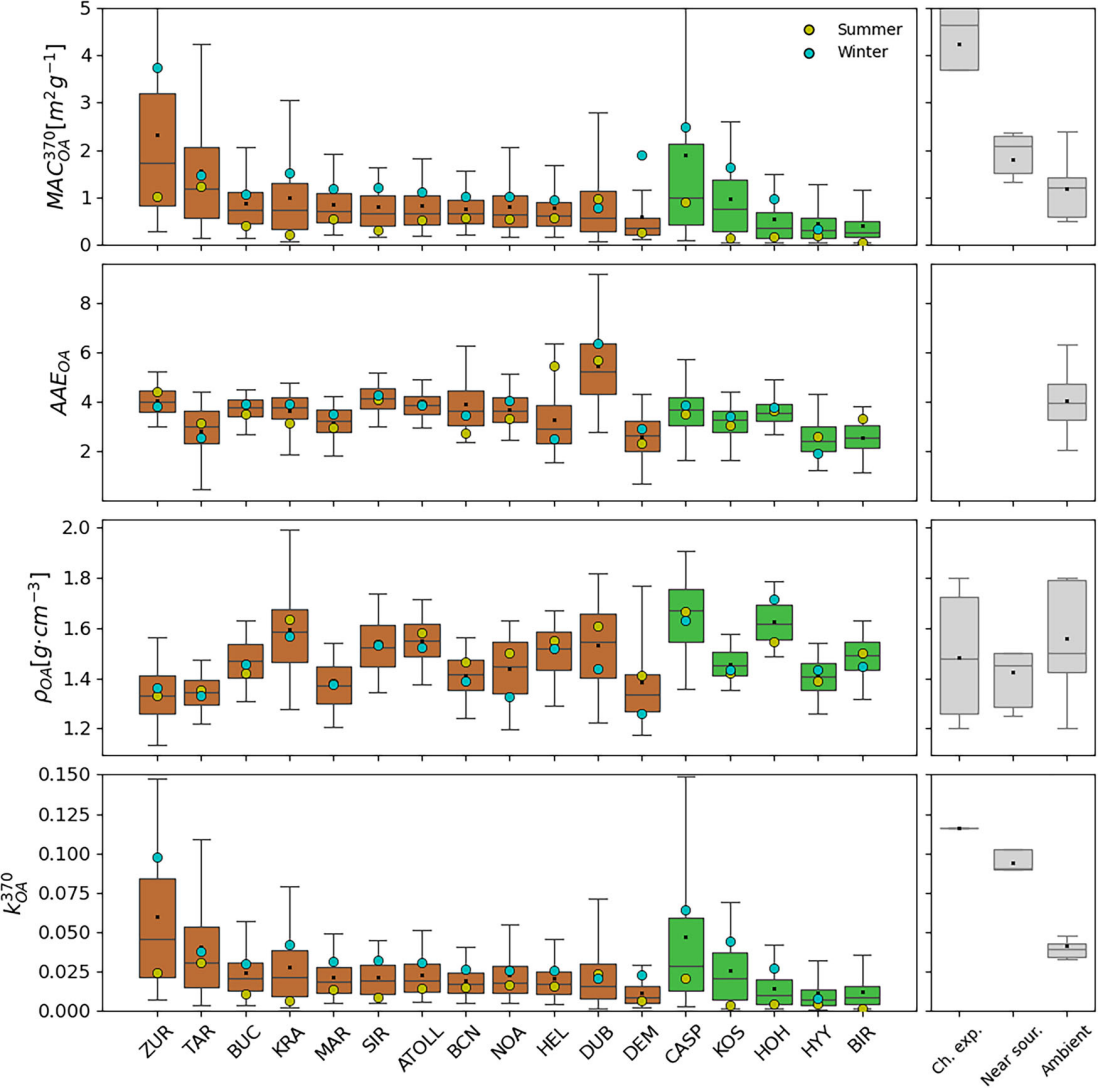
Variartions in BrC optical properties and organic aerosol density across sites. MAC_OA_ and *k*_OA_ at 370 nm, AAE_OA_ (370–590 nm) and density ⍴_OA_ of OA particles studied at the measurement sites used here. Brown and green colors are used for urban and non-urban sites, respectively. Gray box plots on the right represent the range of values published in literature for chamber experiments, field campaigns near the source and ambient measurements (cf. Table S3). Blue and yellow dots represent the mean values of the different variables at each measurement site for winter and summer, respectively.

Figure 2 shows the MAC_OA_ and *k*_*OA*_ at 370 nm, the AAE_OA_ (370–590 nm), and the density of OA particles (*⍴*_OA_) at the measurement sites used here, compared with values reported in the literature (cf. Table S3). Literature values are grouped according to the type of experiment conducted: ambient measurements, as in this work, chamber experiments, and field campaigns near the emission source. Figure 2 shows that the values reported here fall well within the literature range for AAE_OA_ and *⍴*_OA_, while MAC_OA_ and *k*_*OA*_ lie in the lower range. Published values from ambient measurements align more closely with those reported here, whereas MAC_OA_ and *k*_*OA*_ values from chamber experiments and near-source field campaigns are generally higher than those from ambient measurements. This difference reflects the range of conditions—such as fuel type and combustion parameters—achievable in chamber studies, which allow for the investigation of very specific BrC particles. For example, Atwi^44^ reported *k*_OA_ values at 532 nm from biomass combustion chamber experiments that spanned over more than two orders of magnitude, with the lowest *k*_OA_ observed for methanol-soluble BrC and the highest for methanol-insoluble BrC. Moreover, many of these previous studies relied primarily on data from controlled biomass/biofuel combustion experiments or from field observations near strong biomass/biofuel emission sources. As a result, ambient measurements were largely excluded to avoid interference from non-target BrC sources and from SOA. Consequently, one factor contributing to the relatively lower MAC_OA_ and *k*_OA_ values reported in this work, compared to other studies, is the presence of SOA particles, which, as shown previously, exhibit lower absorption efficiency than POA. Figure 2 shows that in winter both MAC_OA_ and *k*_OA_ were higher than in summer and closer to the published values. These higher values in winter were mostly driven by the higher POA/SOA ratios in the cold season (cf. Fig. S7) when the contribution from solid fuel combustion sources (e.g., BBOA and CCOA), with higher MAC, increased^55^. Another possible reason for the difference from literature values obtained in chamber and near-source experiments is the aging of ambient OA particles. These particles are subject to physical processes, such as photobleaching, which can reduce their absorption efficiency after emission or formation. In the case of the AAE_OA_, the data presented here were compared with the AAE_OA_ values recently published by Rovira^6^, based on AE33 measurements from 44 European sites, including those considered in this study. Rovira^6^ presented the biggest collection of BrC AAE_OA_ values for Europe that were obtained using the same procedure used here. Other published BrC AAE_OA_ were often obtained using different wavelength ranges and, consequently, not directly comparable with the values reported in this work. For ⍴_OA_, published values are mostly reported as constants in the literature, whereas here we used ACSM/AMS data to estimate the OA density (cf. “Method” section). Despite the high heterogeneity of the values reported here, these align with the values usually used in literature for OA particles.

Overall, Fig. 2 shows that both MAC_OA_ and *k*_OA_ exhibited higher median values on average at urban sites (0.34–1.73 m^2^g^−1^ and 0.009–0.046, respectively; cf. Table S2) than at non-urban sites (0.27–0.99 m^2^g^−1^ and 0.00–0.029, respectively). We attribute this trend to the higher relative contribution of POA, which has higher MAC (cf. Fig. 1), to OA in urban environments compared to non-urban areas^55^. For the non-urban sites KOS and CASP, the MAC_OA_ (median values 0.76 and 0.99 m^2^g^−1^, respectively) and *k*_OA_ (0.021 and 0.029, respectively) were on average higher compared to BIR, HYY, and HOH and were comparable with the values obtained for urban sites. KOS is surrounded by agricultural land and the most important direct sources of pollution are local roads, domestic heating and a medium-sized timber factory equipped with a biomass furnace that may have contributed to explain the high MAC of OA and of BBOA (cf. Table S2)^67^. At CASP, a strong contribution to PM from marine aerosols has been observed^55,68^. Previous study^6^ suggested that the strong presence of marine aerosol at this site may have affected the aethalometer’s performance by enhancing multiple-scattering artifacts, potentially leading to an overestimation of measured absorption and, consequently, of MAC_OA_^69^.

Among the urban sites, the median MAC_OA_ and *k*_OA_ were highest at ZUR and TAR and lowest at DEM. The elevated MAC_OA_ (1.73 and 1.17 m^2^g^−1^) and *k*_OA_ (0.046 and 0.031) at ZUR and TAR were partly due to the high POA/SOA ratio observed at these sites (not shown), indicating the influence of direct local POA emissions, which enhanced the measured intensive absorption properties. The low MAC_OA_ and *k*_OA_ at DEM (0.34 m^2^g^−1^ and 0.009, respectively), may be attributed to the aging and photobleaching of POA emissions, particularly BBOA, during the transport toward the station^60^. Indeed, as noted above, the MAC of the BBOA source at DEM was the lowest among all sites where BBOA was detected (cf. Table S2). Recently, Kaskaoutis^70^ reported strong eBC emissions, low OC/EC ratio and suppression of AAE values, indicative of lack of BrC, under flaming conditions in close-range fires at the NOA measurement site which is close to the DEM site. Note that the average *b*_Abs,BrC_ (370) and OA mass concentration reported in ref. ^70^ for NOA during August 2021 were around 17.9 Mm^−1^ and 16.3 µg/m^3^, respectively, during burning days, and 3.3 Mm^−1^ and 8.1 µg/m^3^, respectively, during non-burning days, resulting in MAC_OA_ values around 1.1 and 0.4 m^2^g^−1^, comparable with the mean values reported here for NOA and DEM stations. Finally, as reported in Fig. 2, *⍴*_OA_ was on average lower at urban sites (1.32–1.58 g cm^−3^) compared to non-urban sites (1.45–1.68 g cm^−3^), where the OA is likely more aged, resulting in higher ρ_OA_ along with an increase of O:C and the decrease of H:C^71^.

### Parameterizations of MAC_OA_, *k*_*OA*_, *⍴*_OA_, and AAE_OA_ versus log(eBC/OA)

**Fig. 3:**
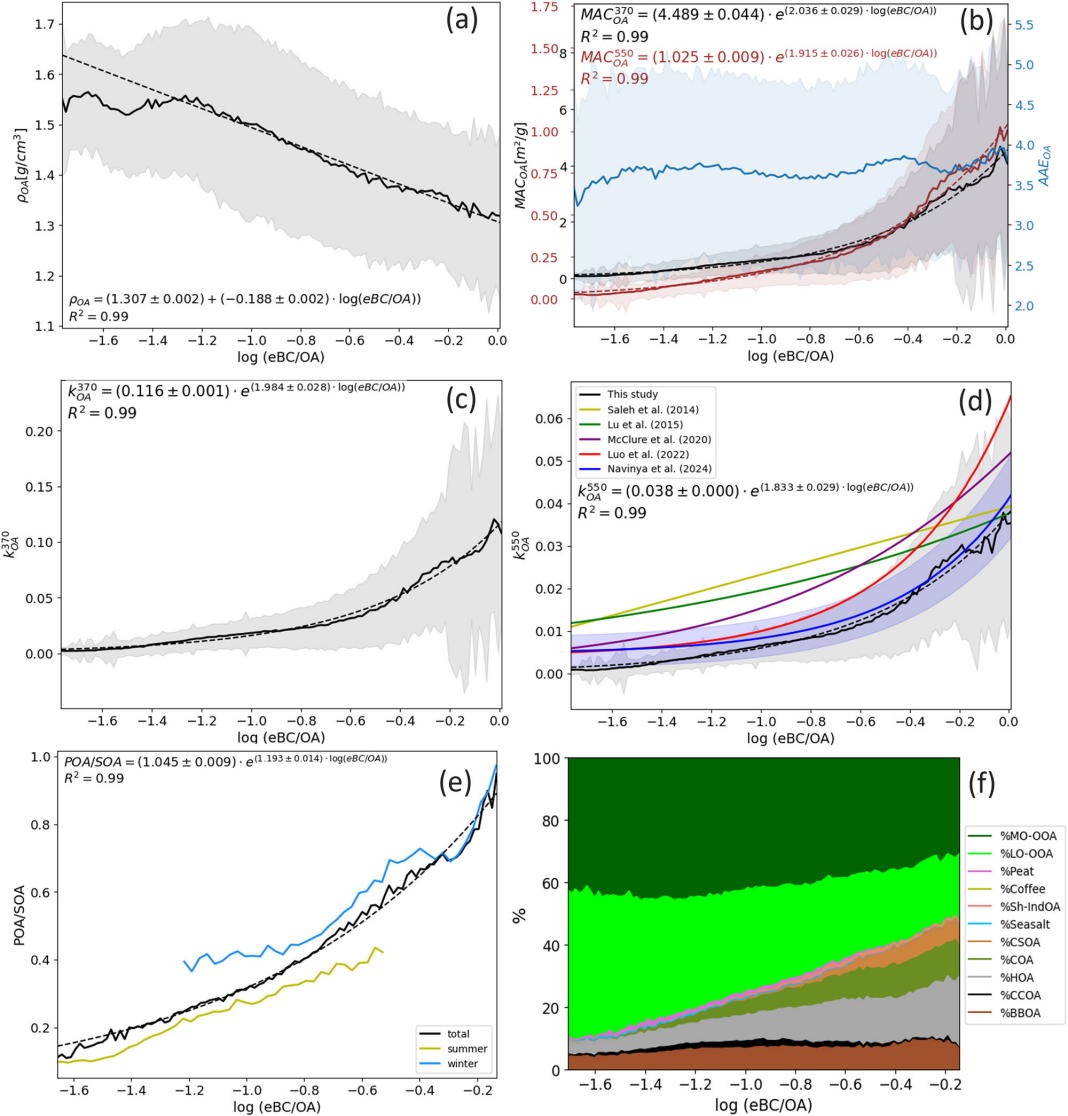
Parameterization of OA optical properties and source contributions as a function of eBC/OA ratio. Dependence of different variables on log(eBC/OA). Black lines represent the binned data and the dashed black lines are the fit. Gray areas represent the standard deviation of the data in this study: **a** OA density *⍴*_OA_ (*⍴*_OA_ = (1.307 ± 0.002) + (−0.188 ± 0.002) · log(eBC/OA)) calculated for −1.3<log(eBC/OA)<0.0; **b** MAC_OA_ at 370 nm (MAC_OA_ = (4.489 ± 0.044) · exp((2.036 ± 0.029) · log(eBC/OA))), MAC_OA_ at 550 nm (MAC_OA_ = (1.025 ± 0.009) · exp((1.915 ± 0.026) · log(eBC/OA))) and AAE_OA_; **c** k_OA_ at 370 nm (*k*_OA_^370^ = (0.116 ± 0.001) · exp((1.984 ± 0.028) · log(eBC/OA))); **d** *k*_*OA*_^*550*^ (*k*_*OA*_^550^ = (0.038 ± 0.000) · exp((1.833 ± 0.029) · log(eBC/OA)); **e** POA/SOA ratio (POA/SOA= (1.045 ± 0.009) · exp((1.193 ± 0.014) · log(eBC/OA))); **f** relative contribution of OA sources to OA at the measurement sites considered in this work. Blue and red areas in Fig. 3b are the standard deviation of AAE_OA_ and MAC at 550 nm values, respectively; red, dark green, light green, purple and blue lines in Fig. 3d are the curves calculated using the parameterizations from Luo^53^, Lu^51^, Saleh^39^, McClure^52^, and Navinya^45^, respectively. The blue area in Fig. 3d is the range of *k*_*OA*_^550^ values based on the parameterization from Navinya^45^; yellow and blue curves in Fig. 3e represent the mean POA/SOA ratio for summer and winter, respectively.

Here, we present parameterizations describing the relationship between *⍴*_OA_, MAC_OA_ (370 and 550 nm), *k*_*OA*_ (370 and 550 nm) and POA/SOA ratio and the ambient eBC/OA ratio (Fig. 3). Figure 3f shows the fractional contribution of POA and SOA sources to bulk OA, while Fig. 3d presents, for comparison, the parametrizations for the *k*_OA_^550^ proposed by previous studies^39,45,51–53^. The trends of these variables are illustrated by the fits shown in each panel. For AAE_OA_ (370–590 nm) the parameterization was not provided as no clear trend between AAE_OA_ and log(eBC/OA) was observed. Figures S3 and S4 show the parameterizations for each individual site for *k*_*OA*_^370^ and AAE_OA_, respectively. Overall, Fig. 3 illustrates a linear decrease in OA density with increasing log(eBC/OA) and an exponential increase in MAC_OA_ and *k*_OA_, reflecting the rise in the POA/SOA ratio, whereas no clear trend was observed for AAE_OA_. The decrease of *⍴*_OA_ with log(eBC/OA) reflected the lower intrinsic densities of OA particles more influenced by combustion (soot-rich) emissions (i.e., high eBC/OA) and consequently compositionally lighter and less oxygenated, leading to a lower ⍴_OA_. Conversely, at low eBC/OA OA is mostly secondary (cf. Fig. 3e, f), enriched in oxygenated functional groups (carbonyl, carboxyl, hydroxyl) and have undergone more aging and oxidation, thus leading to higher ⍴_OA_^71^. Note from Fig. 3a that *⍴*_OA_ remains relatively constant within the range −1.7 < log(eBC/OA) < − 1.3. This corresponds to a very narrow eBC/OA interval (0.02–0.05), and the apparent stability of ⍴_OA_ is amplified by the logarithmic scale. To explore the non-linear behavior of *⍴*_OA_ in this range, f44 and f43 values used to calculate ⍴_OA_ are reported in the Supporting Information as a function of log(eBC/OA) (Fig. S8). As shown in Fig. S8, at low eBC/OA ratios, f44 remains approximately constant, while f43 increases slightly. In this very low log(eBC/OA) range, OA is highly oxidized with minimal primary source contribution, reducing the variability of OA sources and resulting in a relatively stable overall density. Additionally, f43 and f44 may introduce greater uncertainty in this low eBC/OA range. Therefore, the linear fit was restricted to the range −1.3 < log(eBC/OA) < 0.0. Figure 3e, f shows that as the ratio eBC/OA increases, the relative contribution to OA from POA sources originating from primary combustion sources also increases, driving the observed increase of MAC_OA_ (Fig. 3b) and *k*_OA_ (Fig. 3c, d). In fact, as noted above, the highest MAC values were observed for POA sources whereas lower MAC were reported for SOA (cf. Fig. 2). As expected (cf. Eqs. 4 and 10), MAC_OA_ and *k*_OA_ showed similar trends as a function of log(eBC/OA) and reached values close to around 4.5 m^2^/g and 0.12 at 370 nm, respectively, (and 0.04 for the *k*_OA_ at 550 nm) at eBC/OA ratios close to one as typical for fresh biomass burning (e.g., wood or crop residue burning) and fossil fuel combustion sources as diesel engines and coal combustion.

The previously published parameterizations for *k*_*OA*_^*550*^ in Fig. 3d were primarily derived from data obtained in chamber or near-source biomass and biofuel combustion experiments. These studies aimed to investigate the absorption properties of OA particles emitted from biomass/biofuel burning, which are considered among the most important global sources of BrC. OA from these sources is also known to be more strongly absorbing than OA from other primary and secondary sources^5,39^. Furthermore, it has been shown that when data from controlled experiments are used to parameterize *k*_*OA*_^*550*^ versus eBC/OA, then the eBC/OA ratio can be used as an indication of burning conditions^4,5,23,54^.

However, applying these parameterizations in climate models is challenging because emissions inventories typically lack detailed information on burning conditions. In such inventories, the eBC/OA ratio primarily reflects differences in fuel types rather than actual burning conditions^23^. Here, we used ambient data and, consequently, the eBC/OA ratio was driven by different eBC and OA sources and by the relative amount of POA and SOA in OA particles. Figure 3b, c, d shows that the best fit for the MAC_OA_ and *k*_OA_ binned data was represented by exponential functions. It’s worth noting that, despite the observed heterogeneity (i.e., the high standard deviation across all measurement sites), these trends showed a high degree of universality, as exponential increases were observed at all sites considered in this study. (cf. Fig. S3 for *k*_OA_^370^). Overall, a satisfactory agreement for *k*_*OA*_^*550*^ was observed with the parameterizations provided by previous studies^45,53^, that were within the range of variability observed in this study (gray area in Fig. 3d). Over the log(eBC/OA) values reported in Fig. 3d, the parameterizations from previous studies^45,53^ provided *k*_*OA*_^*550*^ values that differed by around 67% (<*k*_*OA*_^550^ > =0.019) and 22% (<*k*_*OA*_^550^ > =0.014), respectively, compared to our parameterization (<*k*_*OA*_^550^ > =0.011). Higher biases were observed with the parameterizations from McClure^52^ (<*k*_*OA*_^550^ > =0.022), Lu^51^ (<*k*_*OA*_^550^ > =0.022) and Saleh^39^ (<*k*_*OA*_^550^ > =0.025), leading to average positive biases of 92%, 99% and 123%, respectively. It is important to note that direct comparison between the parameterizations presented here and those in the literature is challenging for the reasons outlined below. In our study, as the eBC/OA ratio increased, the relative contribution of POA from primary combustion sources to bulk OA also increased (Fig. S3e), unlike in chamber experiments. This trend contributed to the observed increase in *k*_*OA*_^550^, as also reflected by the higher MAC of the POA sources (Fig. 1). Interestingly, better agreement between our parameterization in Fig. 3d and those from Saleh^39^, Lu^51^ and McClure^52^ was observed for eBC/OA values higher than 0.4 (log(eBC/OA) = −0.4) up to around one (log(eBC/OA) = 0). Within this range, a gradual predominance of POA over SOA was observed in the ambient data (cf. Fig. 3e, f), suggesting that the conditions in our measurements more closely resembled those in previous chamber or near-source experiments, where interference from SOA was generally minimized. In this higher range of log(eBC/OA), the parameterizations from Saleh^39^, Lu^51^ and McClure^52^ provided mean *k*_*OA*_^*550*^ values that were higher compared to our values by around 19%, 50%, and 30%, respectively. Indeed, Carter^54^ compared the Saleh^39^ parameterization with experimental estimations of the MAC_OA_ of OA particles measured during three instrumented flights. Carter^54^ reported that the Saleh parameterization overestimated the MAC_OA_ values at 405 nm for flights characterized by low eBC/OA ratios, whereas the comparison improved for flights with higher eBC/OA ratios. This is consistent with the better agreement observed between our data and Saleh^39^ at higher log(eBC/OA). Similarly, Figure S5 shows higher *k*_*OA*_^550^ and a better agreement with the published parameterizations in winter, when a higher POA/SOA ratio was also observed, compared to summer (cf. Fig. 3e). Recent modeling studies have also highlighted that although the Saleh parameterizations are highly valuable for modeling purposes, they may not fully represent actual ambient conditions^35,72^. Another reason for the discrepancy with the literature parameterizations in Fig. 3d might be due to the different techniques used to determine the *k*_*OA*_^550^ of OA particles and the eBC mass concentrations used. For example, Navinya^45^ used EC measurements as a proxy for eBC, Luo^53^ used eBC data from the AE33, whereas Saleh^39^ and McClure^52^ used the mass closure (experimental eBC size distribution data and Mie theory). Other reason for discrepancy in Fig. 3d can be linked with the different log(eBC/OA) ranges used for the parameterization among the published studies, thus limiting the extrapolation of published parameterizations to the average log(eBC/OA) range used here (−1.8 < log(eBC/OA) < 0.0). The log(eBC/OA) ranges reported in previous studies were, for example, −2.4–0 (Saleh^39^), −5.0–0.3 (McClure^52^), −3.4–0 (Lu^51^), −4.0–0 (Saleh^5^), −1.3–0.15 (Luo^53^), −2.0–0 (Navinya^45^). In fact, Shen^72^ commented that the range of log(eBC/OA) ratios in their study (−2.15– −1.21) was on the very small end of the range used in Saleh^39^, and, consequently, the Saleh parameterization failed to capture the absorbing aerosol properties reported by Shen^72^.

Overall, the literature shows a consistent relationship between the *k* Angstrom exponent (the w) and log(eBC/OA). Specifically, although the reported w-log(eBC/OA) trends exhibit considerable variability, they indicate higher wavelength dependence (i.e., higher w) for less absorbing BrC particles—such as SOA with low *k*_OA_ at low eBC/OA—compared to more strongly absorbing BrC^39,44,45,51–53^. For example, Cheng^73^ observed an inverse relationship between AAE_OA_ and EC/OC ratio using data from chamber experiments. They concluded, in agreement with other studies, that the combustion conditions primarily control the AAE_OA_-log(eBC/OA) trend, rather than fuel type or subsequent atmospheric processes. Similarly, Saleh^74^ reported an inverse relationship between w and log(eBC/OA) for OA from biomass burning. They found that SOA produced in aged biomass-burning emissions can be absorptive, exhibiting lower MAC_OA_ and stronger wavelength dependence (i.e., higher w) compared to POA. In our study, no clear relationship between AAE_OA_ and log(eBC/OA) was observed when considering all available data (see Fig. 3b). Indeed, Figure S4 shows that at some sites AAE_OA_ decreased with increasing log(eBC/OA), whereas at other sites it remained nearly constant or even increased. The observed heterogeneity in AAE_OA_ trends was likely due to the diversity of sources (not limited to biomass burning), as well as the varying processes and burning conditions that influence the formation of ambient OA particles. Thus, unlike controlled experiments that focus on the w-log(eBC/OA) relationship for specific OA particles, the complex composition of ambient OA led to the diverse AAE_OA_-log(eBC/OA) trends observed here (Fig. S4). For example, in our study the brownness of ambient OA was driven by the relative amount of POA and SOA originating from multiple sources with SOA (i.e., MO-OOA and LO-OOA) often being non-absorbing (i.e., MAC_OA_ = 0; cf. Table S2). Thus, the absence of absorbing SOA at some sites, combined with the multiple sources of SOA, likely contributed to the lack of correlation between AAE_OA_ and log(eBC/OA) when averaging across all data. Figure S6 shows the AAE_OA_ versus log(eBC/OA) relationship separated for winter and summer. Figure S6b also reports the AAE_OA_ calculated for a short (1.5 months) winter measurement campaign performed in Manlleu (NE Spain) in winter 2016^75^. Manlleu is a small town where fresh and aged BB emissions from domestic, commercial, and agricultural sources were the dominant sources of OA particles in winter. Canals-Angerri^75^ reported that the proportions of BBOA and SOA from BBOA fractions in Manlleu were higher than in most studies^55^, indicating BB as the dominant source of OA. As reported in Fig. S6b, a slight decrease of AAE_OA_ with increasing log(eBC/OA) was observed in winter using all the available data (black line) whereas no clear trend was reported for summer (cf. Fig. S6a). The observed decrease of AAE_OA_ in winter was likely driven by an increased relative importance of BB emissions, and the presence of more absorbing POA, during the cold season at the majority of the measurement sites used here. Moreover, Fig. S6b shows that the AAE_OA_ decrease was very pronounced in Manlleu where SOA was heavily affected by BBOA and, consequently, more absorbing^20,76^. Thus, the data collected in Manlleu agree with the hypothesis that BB emissions can play an important role in dictating an inverse AAE_OA_-log(eBC/OA) relationship. However, as aforementioned, the complexity of ambient OA particles in terms of sources and formation processes prevented us from observing a clear AAE_OA_*-*log(eBC/OA) compared to previous studies targeting BB emissions or specific OA particles.

## Discussion

In this study, we investigated the absorption properties of surface ambient OA particles using ACSM/AMS and AE33 data collected at 17 European measurement sites. We presented the MAC_OA_ and its wavelength dependence (AAE_OA_), the density (⍴_OA_) and the *k*_*OA*_, along with the MAC of various primary and secondary OA sources. We also compared our results with recently published estimates of the same quantities. Consistent with previous research, we propose a series of parameterizations describing the relationships between MAC_OA_, AAE_OA_, ⍴_OA_ and *k*_*OA*_ with the ambient measurements of the eBC-to-OA ratio. This study fills the research gap regarding the representation of the absorption properties of OA particles in climate models. In fact, our analyses are based on ambient measurements, whereas most of the previous works focused on highly-absorbing OA emitted from biomass burning using data from either chamber experiments or close-to-the-source field campaigns. Those studies show substantial heterogeneity, with *k*_OA_ values spanning over more than two orders of magnitude depending on the burning conditions, the fuel type and the type of BrC examined (e.g., water or methanol-soluble/insoluble BrC). We demonstrate that, although chamber experiments are extremely valuable for modeling, they may not fully capture the absorption properties of OA under ambient conditions. A key strength of our approach is that ambient measurements of eBC and OA are widely available in global databases (e.g., EBAS, ebas.nilu.no) and their mass concentrations are routinely simulated by climate models. Therefore, using these data and following the framework here presented, estimations of MAC_OA_, AAE_OA_, *⍴*_OA_, and *k*_OA_ of ambient OA particles in Europe could be performed. However, the parameterization may not be fully representative of unsampled regions, so the results should be carefully evaluated and validated using a similar methodology elsewhere.

Overall, our estimates of AAE_OA_ and ⍴_OA_ fall well within the ranges reported in the literature, while MAC_OA_ and *k*_*OA*_ lie toward the lower end and agree more closely with previous estimates based on ambient measurements. Published MAC_OA_ and *k*_*OA*_ values from chamber experiments and near-source field campaigns tend to be higher, reflecting the wide range of conditions (e.g., BrC sources and combustion settings) that can be reproduced in controlled environments. Moreover, many of these studies focused on biomass burning, which is known to emit BrC particles with higher absorption efficiencies than other primary and secondary OA sources. In our work, the relatively lower MAC_OA_ and *k*_OA_ values are largely driven by the relative contributions of POA and SOA in ambient OA. The presence of low- or non-absorbing SOA effectively “bleaches” the bulk ambient OA compared with source-specific controlled experiments. In fact, here we report the highest MAC values for biomass burning OA (BBOA; 3.52 ± 1.72 m² g⁻¹) and coal combustion OA (CCOA; 4.66 ± 1.87 m² g⁻¹), followed by other POA sources such as traffic (HOA; 1.17 ± 1.07 m² g⁻¹) and cooking (COA; 0.95 ± 0.60 mg⁻¹). Lower MAC values were found for LO-OOA (0.80 ± 0.67 m² g⁻¹) and MO-OOA (0.32 ± 0.21 m² g⁻¹).

Overall, we found that MAC_OA_ (and the *k*_OA_) increased exponentially with the eBC/OA ratio, reflecting the corresponding exponential increase in the POA/SOA ratio as eBC/OA rises. This exponential relationship between MAC_OA_ and *k*_*OA*_ versus log(eBC/OA) was observed consistently across all sites, confirming the enhancement of OA absorption with increasing eBC/OA reported in the literature, regardless of whether measurements were collected under ambient or controlled conditions.

However, as noted earlier, in controlled experiments the observed trends are primarily dictated by combustion conditions—and, to a lesser extent, fuel type—whereas in our ambient dataset the trends are mainly driven by changes in the POA/SOA ratio. The winter parametrization derived here for the *k*_*OA*_^550^– log(eBC/OA) relationship aligns more closely with previously published parameterizations, particularly for moderately to highly absorbing OA. Under these conditions, ambient OA is likely more similar to the OA typically investigated in controlled experiments, contributing to the stronger agreement. In contrast, during summer, the lower POA/SOA ratio—resulting from higher relative concentrations of low- or non-absorbing SOA—led to substantially lower *k*_*OA*_ values compared with previously reported parameterizations.

Unlike MAC_OA_ and *k*_*OA*_, we did not observe a clear relationship between AAE_OA_ and log(eBC/OA). Previous parameterizations have reported a stronger wavelength dependence for low-absorbing SOA than for more absorbing OA. This is primarily because low-absorbing SOA absorbs mainly in the UV, and its absorption decreases rapidly with increasing wavelength, which results in higher AAE_OA_ and higher *w*. In contrast, our ambient dataset reflects a complex mixture of OA sources—both absorbing and non-absorbing—as well as varying degrees of aging and mixing with other chemical species. Consequently, AAE or *w* represent effective bulk properties of the overall particle mixture rather than isolated SOA or POA contributions. Interestingly, our data show an inverse relationship between AAE_OA_ and log(eBC/OA) during winter, likely due to the increased influence of biomass burning and POA emissions. A clear decrease in AAE_OA_ with increasing log(eBC/OA) was observed in the small village of Manlleu (NE Spain), where fresh (POA) and aged (SOA) biomass burning emissions account for up to more than 30% of OA in winter. This supports the idea that biomass burning emissions and the formation of absorbing SOA drive the inverse *w*–log(eBC/OA) relationships reported in the literature.

Finally, we found a linear decrease in ⍴_OA_ with increasing eBC/OA ratio, reflecting the lower densities of OA particles that are more strongly influenced by combustion emissions (i.e., at high eBC/OA). Conversely, at low eBC/OA, OA is predominantly secondary, enriched in oxygenated functional groups, and has undergone more extensive aging and oxidation, which leads to higher ⍴_OA._

In conclusion, we have presented and discussed the first comprehensive European dataset describing the intensive absorption properties of ambient OA particles based on surface monitoring measurements. This work complements previous phenomenological studies that reported OA mass concentrations and absorption properties across Europe. Chen^55^ characterized OA mass concentrations and source apportionment using ACSM/AMS data and a state-of-the-art source apportionment protocol across 22 sites. Rovira^6^ used multi-wavelength aethalometer measurements from 44 European sites to examine regional and seasonal variability, as well as long-term trends, in the absorption coefficients of carbonaceous aerosols (eBC and BrC). Here, we integrate these sources of information to advance the characterization of OA absorption properties. Specifically, we present MAC, AAE, density, and *k* values for OA particles at the 17 measurement sites common to both Chen^55^ and Rovira^6^. Because the parameterizations developed in this study are derived from ambient measurements on a continental scale, they are well-suited for use in climate models to better constrain the absorptive properties of bulk OA, provided that ambient OA and eBC mass concentrations are known. By providing parameterizations grounded in real ambient observations across Europe, this study offers policymakers and society more reliable constraints on the climate impacts of organic aerosol, ultimately supporting the development of more effective air-quality and climate-mitigation strategies.

## Methods

### Measurements sites and instruments

OA mass concentrations and aerosol particles multi-wavelength absorption coefficients were measured at 17 sites in Europe by means of ACSM/AMS instruments (14 quadrupole ACSM, 2 Time-of-Flight ACSM, 1 Compact Time-of-Flight AMS) and Aethalometers (model AE33, Aerosol Magee Scientific), respectively. The 17 sampling sites were classified following the classification from Chen^55^ as urban (12 sites, including four flagged as suburban: DEM, ATOLL, SIRTA, and BUC) and non-urban (5 sites) (Table S1).

### Data collection and data treatment

ACSM and AMS data were aggregated within the framework of the Chemical On-Line cOmpoSition and Source Apportionment of fine aerosoL (COLOSSAL) project (https://www.cost.eu/actions/CA16109/), AE33 were collected within the EU FOCI (https://www.project-foci.eu/wp/) and RI-URBANS (https://riurbans.eu/) projects. OA mass concentrations data and OA source contributions used here were published in Chen^55^. We refer to Chen^55^ and references therein for a detailed description of ACSM/AMS working principle, uncertainties and data collection at the measurement sites in Table S1. Considering the objectives of this study, we provide additional context to Chen^55^ regarding potential sources of error, specifically the effect of insoluble, refractory dark-BrC on measurements. Refractory tar balls from biomass burning could, in principle, lead to underestimation of BBOA mass by ACSM/AMS. However, systematic mass-closure procedures and the COLOSSAL standard operating procedure (SOP) align ACSM/AMS data with independent PM_1_ measurements using appropriate collection efficiencies, compensating for missing refractory mass^55,77^. Moreover, the data in this study come from European sites dominated by residential biomass burning, where tar-ball formation is expected to be low. Consistently, Moschos^43^ found insoluble tar balls negligible in wintertime residential wood-burning aerosol in Switzerland, with short-wavelength NR-PM absorption attributed entirely to methanol-extractable BrC. Therefore, the measurement-based BBOA reported by Chen^55^, to the authors’ knowledge, represents the most reliable estimate of brown carbon to date. Based on these considerations, the impact of tar balls on the absorption measurements used here can be regarded as minimal.

Black carbon (eBC) mass concentrations and aerosol particles light absorption at seven different wavelengths (370, 470, 520, 590, 660, 880, and 950 nm) were derived from AE33 instruments. As any other filter-based absorption photometer, the AE33 is subject to some artifacts related to the presence of the filter tape. As explained below, we corrected eBC and absorption coefficients using the latest harmonization procedure available from the Aerosol, Clouds and Trace Gases Research Infrastructure (ACTRIS; https://www.actris.eu/)^78,79^. An extensive description of the AE33 was provided by Drinovec^48^. The absorption coefficient (*b*_Abs_ (*λ*)) (in Mm^−1^) were retrieved from the raw equivalent black carbon (eBC) mass concentrations following Eq. 1^78–80^:

1 $${b}_{{Abs}}\left(\lambda \right)={BC}(\lambda )\,\begin{array}{l}\cdot \,\end{array}\frac{{MA}{C}_{{BC}}\left(\lambda \right)}{H* },$$

where H* is the ACTRIS harmonization factor that, as commented below, depends on the filter tape used and it is 2.21 for the M8020 and M8050 filter tapes and 1.76 for the M8060 filter tape^78^. The AE33 software applies a correction factor (*C*) to account for multiple scattering by filter fibers, with values of 1.39 for the current M8060 tape and 1.57 for older tapes (M8020/M8050). However, studies have shown that *C* increases when particles accumulate on the filter, ranging 2.2–3.4 for M8060 and 2.3–4.2 for older tapes, reflecting particle penetration and scattering effects^78,81–84^. These studies provided a mean *C* value for M8060 tape of around 2.6 ± 0.3, closely matching the ACTRIS-recommended value of 2.44. and of 3.0 ± 0.5 for the older tapes. Extreme values up to ~5 have been observed under specific conditions, such as pure dust^69,85^, lab-generated soot (<200 nm), or highly scattering particles with a single scattering albedo higher than 0.95^81,86^. Since experimental determination of C requires comprehensive measurements, which are unavailable at most sites considered here, we adopted the aforementioned ACTRIS-recommended C values to harmonize absorption measurements and to ensure consistent AE33 data reporting across Europe^79,80^. Consequently, in Eq. 1, H* is obtained from the ratio of the ACTRIS-recommended *C* values and the *C* values adopted by the AE33 software. Based on the aforementioned studies, a 15–20% uncertainty should be associated with the H* values adopted here. In Eq.1, MAC (*λ*) is the default mass absorption cross section in m^2^/g at the seven wavelengths from 370 to 950 nm used by the AE33 software. Recently, Savadkoohi^79^ reported a median value for Europe for the MAC at 880 nm of 7.83 m^2^/g, close to the default value of 7.77 m^2^/g used in the AE33 software. The harmonized eBC mass concentrations were then calculated by dividing the harmonized b_Abs_(*λ*) by the MAC at 880 nm. The AE33 instruments deploy a real-time filter loading effect compensation algorithm based on the dual spot technology^48^. The AE33 data were averaged over the ACSM data timestamp (30 min).

### Estimation of BrC contribution to absorption

The contribution of BrC (*b*_Abs,BrC_ (*λ*)) to the total measured absorption (*b*_Abs_ (*λ*)) at different wavelengths from 370 nm to 660 nm was estimated by subtracting the absorption due to eBC (*b*_Abs,BC_ (*λ*)) to the measured *b*_Abs_ (*λ*) (Eq. 2 and Eq. 3), assuming that BrC particles do not absorb in the near-IR (880 and 950 nm)^6,18,20,49,50^.

2 $${b}_{{Abs},{BC}}\left(\lambda \right)={b}_{{Abs}}\left(880\,{nm}\right){\left(\frac{880}{\lambda }\right)}^{{AA}{E}_{{BC}}}$$

3 $${b}_{{Abs},{BrC}}\left(\lambda \right)={b}_{{Abs}}\left(\lambda \right)-{b}_{{Abs},{BC}}\left(\lambda \right)$$

The AAE_BC_ in Eq. 2 represents the AAE of eBC particles and was estimated at each site following the procedure described in previous studies^6,35,87^. Specifically, it was defined as the 1st percentile of AAE values calculated from 370 to 950 nm. This is the common wavelength range used for the AAE calculation, as eBC efficiently absorbs across the near-infrared wavelengths as well. Because BrC increases AAE, the 1st percentile approximates the lowest AAE values corresponding to eBC-dominated absorption coefficients. To reduce the influence of AAE values with high signal-to-noise ratios, the 1st percentile was calculated only from spectral fits with R2 > 0.99^6,88^. The resulting 1st percentile values (see Fig. S1) yielded AAE_BC_ estimates ranging from 0.90 to 1.11, consistent with the range of AAE values typically associated with eBC from fossil fuel combustion^89,90^. Two exceptions were the Irish measurement sites DUB and CASP, where the 1st percentile produced low AAE_BC_ values (0.67 and 0.87, respectively). CASP and DUB are sites where a strong contribution of marine aerosols to PM has been observed^55^, which could have considerably increased the single scattering albedo (SSA) of the particles collected on the filter tape. Elevated SSA may influence *b*_abs_ (*λ*) and, consequently, the calculated AAE^49,69^. For these sites, an AAE_BC_ value of 1—commonly assumed in previous studies—was adopted^6,34,50,91–93^.

It should be noted that Eqs. 1 and 2 are applicable provided that the contribution of dust particles to light absorption is negligible. The sporadic contribution of dust to absorption measured by filter-based absorption photometers has been isolated in previous studies either at remote sites, where the background absorption due to eBC or BrC is on average very low, or during field experiments conducted in dust source regions with active dust emissions^69,94–97^. Conversely, the dust contribution to absorption is generally considered as minimal at sites dominated by carbonaceous aerosols. The mass absorption cross-section (MAC) of Saharan dust is substantially lower than that of eBC or BrC, with estimates for North African desert dust ranging from 0.1 to 0.24 m² g⁻¹ at 370 nm^98^. While the transport of large amounts of dust potentially affecting the absorption cannot be excluded, these events are sporadic, resulting in short-term impacts on absorption. Thus, their long-term effect at sites dominated by carbonaceous aerosols is generally considered negligible^79,80^. For example, Savadkoohi^79^ compared absorption and elemental carbon (EC) measurements at the DEM site in Greece (also included in this study) and showed that the slopes of absorption versus EC scatter plots (i.e., the MAC) were very similar whether dust-affected days were included or excluded, indicating that excluding dust events does not substantially alter the obtained MAC values. Similarly, Savadkoohi^80^ found that Saharan dust outbreaks at the Barcelona site (BCN, also used here) had a minimal impact over long timescales, as evidenced by the strong correlation between eBC and NO₂ concentrations. Therefore, considering that most of the measurement stations included here are urban or suburban, and that the more remote ones (e.g., Birkenes, HOH, HYY) are far from dust source regions, we can reasonably exclude a significant effect of dust on the long-term measurements presented.

### Calculation of the physical properties of OA particles

In the following, we describe the methodology used to calculate the physical properties of OA particles, i.e., the mass absorption cross section (MAC_OA_), the MAC_OA_ Ångström exponent (AAE_OA_), the density of OA particles (*⍴*_OA_) and the imaginary refractive index *k*_OA_ (Eqs. 4–10):

4 $${MA}{C}_{{OA}}(\lambda )=\frac{{b}_{{Abs},{BrC}}(\lambda )}{{OA}},$$

5 $${AA}{E}_{{OA}}=-\frac{ln\,({MA{C}_{\lambda }}_{1}/{MA{C}_{\lambda }}_{2})}{ln\,({\lambda }_{1}/{\lambda }_{2})},$$

where AAE_OA_ was calculated over the 370–590 nm range. The BrC absorption at the long-visible wavelength (i.e., 660 nm) was very low at some of the measurement sites included in this study and was therefore excluded from the calculation of AAE_OA_ to avoid introducing undesired noise and to ensure a consistent comparison of AAE_OA_ across all sites using a common wavelength range. Note from Eq. 5 that the AAE_OA_ of the MAC_OA_ (Eq. 4) is equal to the AAE_OA_ of ${b}_{{Abs},{BrC}}\left(\lambda \right)$. The OA density, *⍴*_OA_, was calculated using Eq. 6^99^:

6 $${\rho }_{{OA}}=\frac{12+H:C+16{\rm{\cdot }}O:C}{7+5{\rm{\cdot }}H:C+4.15{\rm{\cdot }}O:C},$$

where the oxygen-to-carbon (O:C) and hydrogen-to-carbon (H:C) atomic ratios were obtained following Eqs. 7 and 8^100^.

7 $$H:C=1.12+6.74\,{\rm{\cdot }}\,{f}_{43}-17.77\,\cdot \,{({f}_{43})}^{2}$$

8 $$O:C=0.079+4.31\,\cdot \,{f}_{44}$$

Recently, Poulain^101^ reported that using *f*_44_ from the ACSM to estimate the atomic O:C ratio (Eq. 8) should be approached with caution, due the large variability of *f*_*44*_ signal. This variability has been attributed to instrument-dependent differences in vaporization conditions and/or possible matrix effects^102^. Consequently, Poulain^101^ applied the same approach previously developed for the AMS by Canagaratna^100^ to estimate OC concentrations from the ACSM (OC_ACSM_; Eq. 9) and compared these values with OC obtained from offline filter analysis (OC_PM_).

9 $${OA}:O{C}_{{ACSM}}=1.29\,\cdot \,O:C+1.17$$

Poulain^101^ reported slope, intercept and *R*^2^ of 0.65, 0.26 µg/m^3^ and 0.73, respectively, by comparing OC_ACSM_ with OC_PM1_, whereas a lower slope of 0.42 was reported comparing with OC_PM2.5_ due to the presence of OC in the PM_1-2.5_ fraction. Based on the OC_ACSM_ vs OC_PM1_ comparison, Poulain^101^ concluded that their ACSM provided a relatively realistic value of the *f*_44_ and, consequently, a reasonable proxy for the O:C and OM:OC ratios. Poulain^101^, also emphasized the need for further systematic comparisons between OC_ACSM_ and collocated OC_PM1_, noting that a similar approach could yield different results with other ACSMs or in different locations. Furthermore, they highlighted the importance of the filter sample size cutoff in such comparisons, which typically use PM₂_._₅, for which a standardized thermal–optical analysis method (EUSAAR II) is available^103^. To assess the feasibility of using the ACSM *f*_*44*_ in this study, we applied Eq. 9 to a subset of sites (8 sites) for which PM filter analysis was available and compared the obtained OC_ACSM_ with OC_PM1_ at BCN, KRA and MAR and with OCPM2.5 at BIR, SIRTA, DEM, KOS and NOA. The instruments used for this test were five Q-ACSM, two ToF-ACSM and one C-ToF-AMS and the results of the comparison were reported in Fig. S2. For the calculations, only *f*_*44*_ values above the detection limits of the instruments were used^104–106^. OC_ACSM_ data in Fig. S2 were averaged over 24 h to match the OC_PM_ time stamp and points with data coverage lower than 75% were removed from the analysis. Moreover, at KOS, where the C-ToF-AMS was deployed, winter data were excluded due the poor correlation with OC_PM_, whereas no significant seasonal differences were observed at the other sites. The reasons for the poor correlation in winter at KOS are unknown but could be related to the different types of aerosol in winter at this site, where anthropogenic aerosol from heating dominates^107^, and to the fact that AMS instruments measure higher masses compared to ACSM which are typically from combustion sources.

As shown in Fig. S2, the comparison between OC_ACSM_ and OC_PM1_ yielded slopes ranging from 0.997 to 1.141, intercepts from −0.183 to 0.754, and *R*² values between 0.77 and 0.79. Therefore, consistent with Poulain^101^, we concluded from this analysis that the instruments used at BCN, MAR, and KRA provide realistic f44 values that can be used as proxies for the O:C and OM:OC ratios. Similar conclusions can be drawn for the other five sites, where the comparison was made with OC_PM2.5_. In these cases, the average slope, intercept, and *R*^2^ were 0.97 ± 0.19 (from 0.71 to 1.16), −0.34 ± 0.56 (from −1.20 to 0.28), and 0.81 ± 0.13 (from 0.64 to 0.95), respectively. However, we cannot rule out the presence of OC in the PM_1–2.5_ fraction at these sites. Several previous studies have consistently reported that PM_1_ OC represents on average around 80% of PM_2.5_ OC^108–113^. Assuming that OC_PM2.5_ is approximately 20% higher than OC_PM1_, the slopes reported above would increase by roughly the same amount, yielding values around 1.17 ± 0.23 (ranging from 0.85 to 1.39), which are comparable to, or even better than, those reported by Poulain^101^.

Consequently, given the overall good agreement in the reported comparisons (cf. Fig. S2), we applied Eqs. 6–8 to all sites to estimate the OA density. Moreover, a brief sensitivity test showed that an uncertainty of ±40% in *f*_44_ led to an uncertainty of ±10% in ⍴_OA_ when using Eqs. (6), (7) and (8). This level of uncertainty is acceptable and falls within the overall uncertainty range.

Finally, we estimated the *k*_OA_ at 370 nm with Eq. 10 using the MAC_OA_/MAE_OA_ ratio at 370 nm provided by Moschos^43^.

10 $${k}_{{OA}}=\frac{{\rho }_{{OA}}\,\cdot \,\lambda \,\cdot \,{MA}{C}_{{OA}}\,\cdot \,{({MA}{C}_{{OA}}/{MA}{E}_{{OA}})}^{-1}}{4\pi }$$

It is common practice to use the MAC_OA_/MAE_OA_ ratio to estimate the *k*_OA_ once MAC_OA_ is known^43,114,115^. In the literature, a constant MAC_OA_/MAE_OA_ ratio of 1.8–2.0 is frequently applied to proceed from absorption measured in dilute solutions to absorption in the particle phase^116–118^. However, Moschos^43^ has shown that MAC_OA_/MAE_OA_ ratios are not constant and can be lower than those used in the literature. They reported total BrC MAC_OA_/MAE_OA_ values ranging between 1.3 and 1.6, depending on the size range considered and mixing state with inorganic components, and factor-specific values from around 1.3 for primary OA (e.g., BBOA; HOA and COA) to 1.6−1.7 for less absorbing SOA. Since we lack MAE_OA_ measurements on the available data, we used the MAC_OA_ from Eq. 4 together with the MAC_OA_/MAE_OA_ ratio at 370 nm for different OA sources presented in Moschos^43^ to estimate the *k*_OA_ at 370 nm. For this, we used the OA source contributions presented in Chen^55^ for the 17 measurement sites used here. In order to obtain a representative MAC_OA_/MAE_OA_ ratio for total OA, we weighted the factor-specific MAC_OA_/MAE_OA_ at 370 nm from Moschos^43^, considering the fractional contribution of each source to OA.

Since the parameterizations describing the relationships between OA absorption properties and the eBC/OA ratio are available in the literature for the *k*_OA_ at 550 nm, we used Eq. 11 to report the obtained *k*_OA_ at 370 to 550 nm:

11 $${{k}_{OA}}^{\lambda }={{k}_{OA}}^{370}\,\cdot \,{(370/\lambda )}^{\omega }$$

where *w* is the *k*_OA_ Angstrom exponent, calculated as w = AAE_OA_ − 1^45,51^.

### MAC_OA_ of OA sources

The MAC_OA_ of OA sources was obtained through a multilinear regression analysis (MLR; cf. Eq. 12) where the *b*_Abs,BrC_ (*λ*) calculated from Eq. 3 was the dependent (response) variable and the OA source contributions were the independent (predictors) variables. The MLR was applied to *b*_Abs,BrC_ (*λ*) for wavelengths between 370 and 590 nm and the obtained MAC_OA_ (*λ*), in m^2^g^−1^, were then used to calculate the AAE_OA_ for the considered OA sources.

12 $${b}_{{abs},\,{BrC}}\left(\lambda \right)=\mathop{\sum}\limits_{i=1}^{n}{{MA}{C}_{{OA}}}_{n}\left(\lambda \right)\,\cdot \,\left[{{source}}_{n}\right]+{intercept},$$

It should be noted that the MLR yields MAC values that represent a data-driven statistical sensitivity of the total BrC absorption to each OA factor under sampled real-world conditions, rather than intrinsic absorption properties associated with isolated compounds of those sources. Consequently, a weak correlation between any OA source and BrC absorption in the MLR does not necessarily imply a reduced absorptivity, but may instead reflect periods during which BrC absorption is dominated by other sources. For this reason, all data points were included in the MLR, and the resulting MAC values for the OA sources at the considered measurement sites should be interpreted as average MAC across all possible conditions.

## Supplementary information

Supplementary Information

## Data Availability

Supplementary data to this article can be found online at 10.5281/zenodo.17649258.
