# Supplementary material for: Constraining the intensive absorption properties of ambient organic aerosol particles based on pan-European observations
Source: NPJ Clim Atmos Sci. 2026 Apr 10;9(1):131. doi: 10.1038/s41612-026-01405-9 (PMC13253367; doi:10.1038/s41612-026-01405-9)
Supplement: Supplementary file 1 — Supplementary Information [file 41612_2026_1405_MOESM1_ESM.pdf]

Supplemental material: Constraining the intensive absorption properties of ambient organic aerosol particles based on pan-European observations.

Jordi Rovira [a,b,*], Jesús Yus-Díez [c,*], Gang I. Chen [d], Griša Močnik [c,e,f], Martin Gysel-Beer [g], Wenche Aas [h], Minna Aurela [i], John Backman [i], Sujai Banerji [j], Benjamin T. Brem [g], Anna Canals-Angerri [a], Benjamin Chazeau [g,k], Kaspar R. Daellenbach [g], Joel F. de Brito [l], Evangelia Diapouli [m], Konstantinos Eleftheriadis [m], Mikael Ehn [j], Olivier Favez [n], Harald Flentje [o], Maria I. Gini [m], Asta Gregorič [c,p], Roy Harrison [q], Liine Heikkinen [j], Christoph Hueglin [r], Antti Hyvärinen [i], Matic Ivančič [p], Hannes Keernik [s,t], Granakis Konstantinos [m], Eleni Liakakou [u], Chunshui Lin [v], Radek Lhotka [w], Krista Luoma [i,j], Marek Maasikmets [s], Hanna E. Manninen [x], Manousos Ioannis Manousakas [g], Nicolas Marchand [k], Saliou Mbengue [y], Nikos Mihalopoulos [u], María Cruz Minguillón [a], Doina Nicolae [z], Jarkko V. Niemi [x], Jurgita Ovadnevaite [v], Noemí Pérez [a], Jean-Eudes Petit [aa], Stephen M. Platt [h], Petra Pokorná [w], André S.H. Prévôt [g], Véronique Riffault [l], Martin Rigler [p], Matteo Rinaldi [ab], Jaroslav Schwarz [w], Iasonas Stavroulas [c], Erik Teinemaa [s], Kimmo Teinilä [i], Hilkka Timonen [i,ac], Anna Tobler [g,ad], Jeni Vasilescu [z], Marta Via [c], Petr Vodička [w], Stergios Vratolis [m], Karl Espen Yttri [h], Naděžda Zíková [w], Olga Zografou [m], Andrés Alastuey [a], Tuukka Petäjä [j], Xavier Querol [a], and Marco Pandolfi [a,*]

[a] Institute of Environmental Assessment and Water Research (IDAEA-CSIC), 08034 Barcelona, Spain
[b] Department of Applied Physics-Meteorology, Universitat de Barcelona, Barcelona, 08028, Spain
[c] Center for Atmospheric Research, University of Nova Gorica, Ajdovščina, 5270, Slovenia
[d] Environmental Research Group, MRC Centre for Environment and Health, Imperial College London, London W12 0BZ, UK
[e] Haze Instruments d.o.o., Ljubljana, 1000, Slovenia
[f] Department of Environmental Sciences, Jozef Stefan Institute, Ljubljana, 1000, Slovenia
[g] PSI Center for Energy and Environmental Sciences, Paul Scherrer Institute, 5232 Villigen PSI, Switzerland
[h] NILU, 2027 Kjeller, Norway
[i] Atmospheric Composition Research, Finnish Meteorological Institute, 00560, Helsinki, Finland
[j] Institute for Atmospheric and Earth System Research/Physics (INAR), Faculty of Science, University of Helsinki, Helsinki, Finland
[k] Aix Marseille Univ., CNRS, LCE, Marseille, France
[l] IMT Nord Europe, Institut Mines-Télécom, Univ. Lille, Centre for Energy and Environment, Lille, France
[m] ENRACT, Institute of Nuclear and Radiological Science & Technology, Energy & Safety, NCSR Demokritos, 15310 Ag. Paraskevi, Athens, Greece
[n] Institut National de l'Environnement Industriel et des Risques (INERIS), Verneuil-en-Halatte, France
[o] German Meteorological Service (DWD), Observatory Hohenpeissenberg, Germany
[p] Aerosol d.o.o., Kamniška 39A, 1000 Ljubljana, Slovenia
[q] School of Geography, Earth & Environmental Sciences, University of Birmingham, Edgbaston, Birmingham B15 2TT, United Kingdom
[r] Laboratory for Air Pollution and Environmental Technology, Swiss Federal Laboratories for Materials Science and Technology (Empa), Duebendorf, Switzerland
[s] Estonian Environmental Research Centre, Air Quality Management Department, Tallinn, Estonia
[t] Institute of Physics, University of Tartu, Tartu, Estonia
[u] Institute for Environmental Research & Sustainable Development, National Observatory of Athens, Athens, Greece
[v] School of Natural Sciences, Physics, Centre for Climate and Air Pollution Studies, Ryan Institute, University of Galway, University Road, Galway H91 CF50, Ireland
[w] Institute of Chemical Process Fundamentals of the Czech Academy of Sciences, Rozvojová 135/1, 16500 Prague, Czech Republic

[x] Helsinki Region Environmental Services Authority (HSY), 00240, Helsinki, Finland
[y] Global Change Research Institute, Czech Academy of Sciences, 603 00 Brno, Czech Republic
[z] National Institute of Research and Development for Optoelectronics INOE 2000, Magurele, Romania
[aa] Laboratoire des Sciences du Climat et de l'Environnement, CEA/Orme des Merisiers, Gif-sur-Yvette, France
[ab] Institute of Atmospheric Sciences and Climate (ISAC), National Research Council (CNR), 40129 Bologna, Italy
[ac] Aerosol Physics Laboratory, Faculty of Engineering and Natural Sciences, Tampere University, Tampere University, P.O. Box 692, FI-33014, Finland
[ad] Datalystica Ltd., Parkstrasse 1, 5234 Villigen, Switzerland

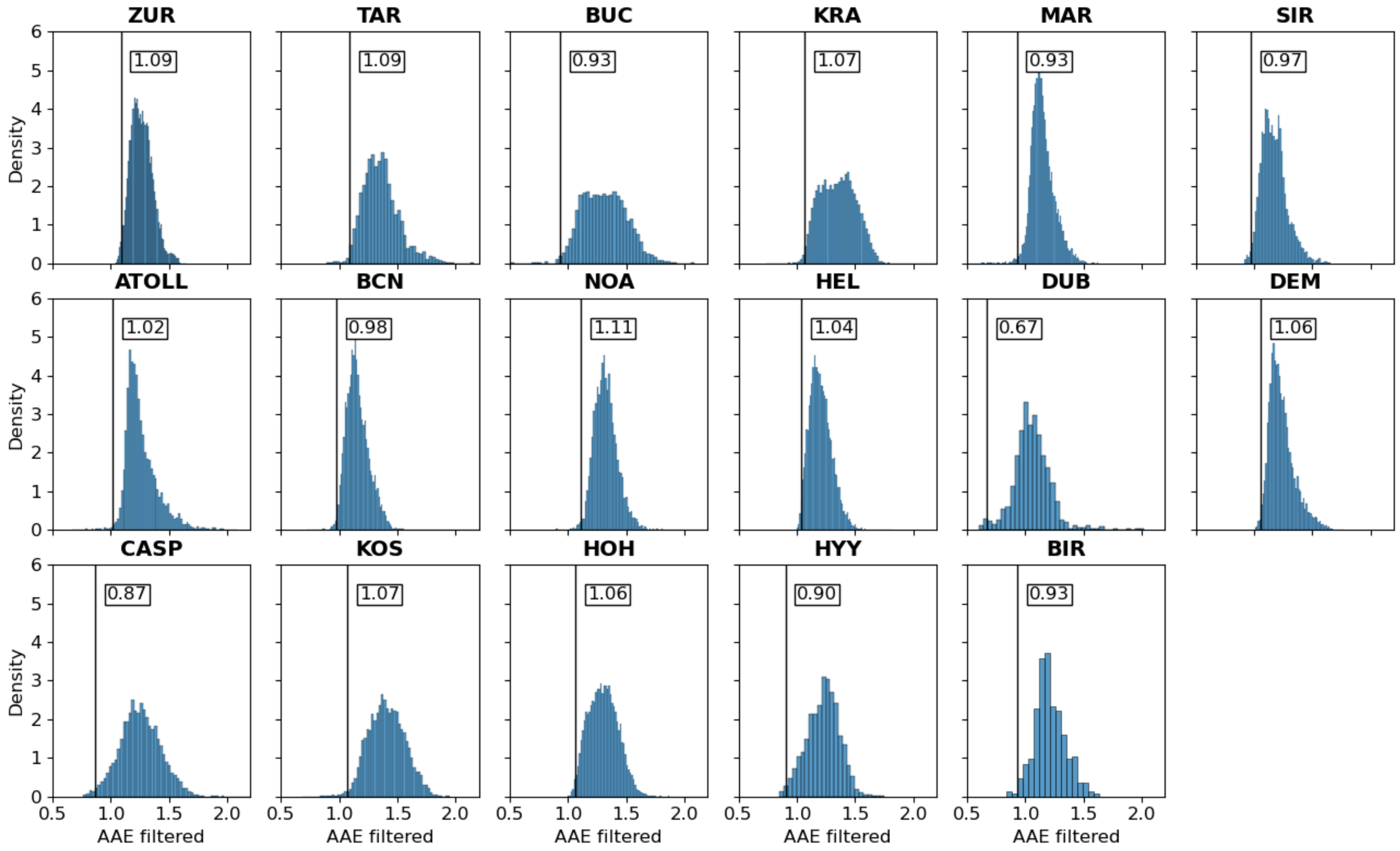

Figure S1: **Site-dependent AAE frequency distributions.** Frequency distributions of AAE (370-950 nm) for the 17 measurement stations. Vertical lines and numbers indicate the 1st percentiles (i.e. the AAE_{BC}) calculated from AAE values filtered by R^2>0.99.

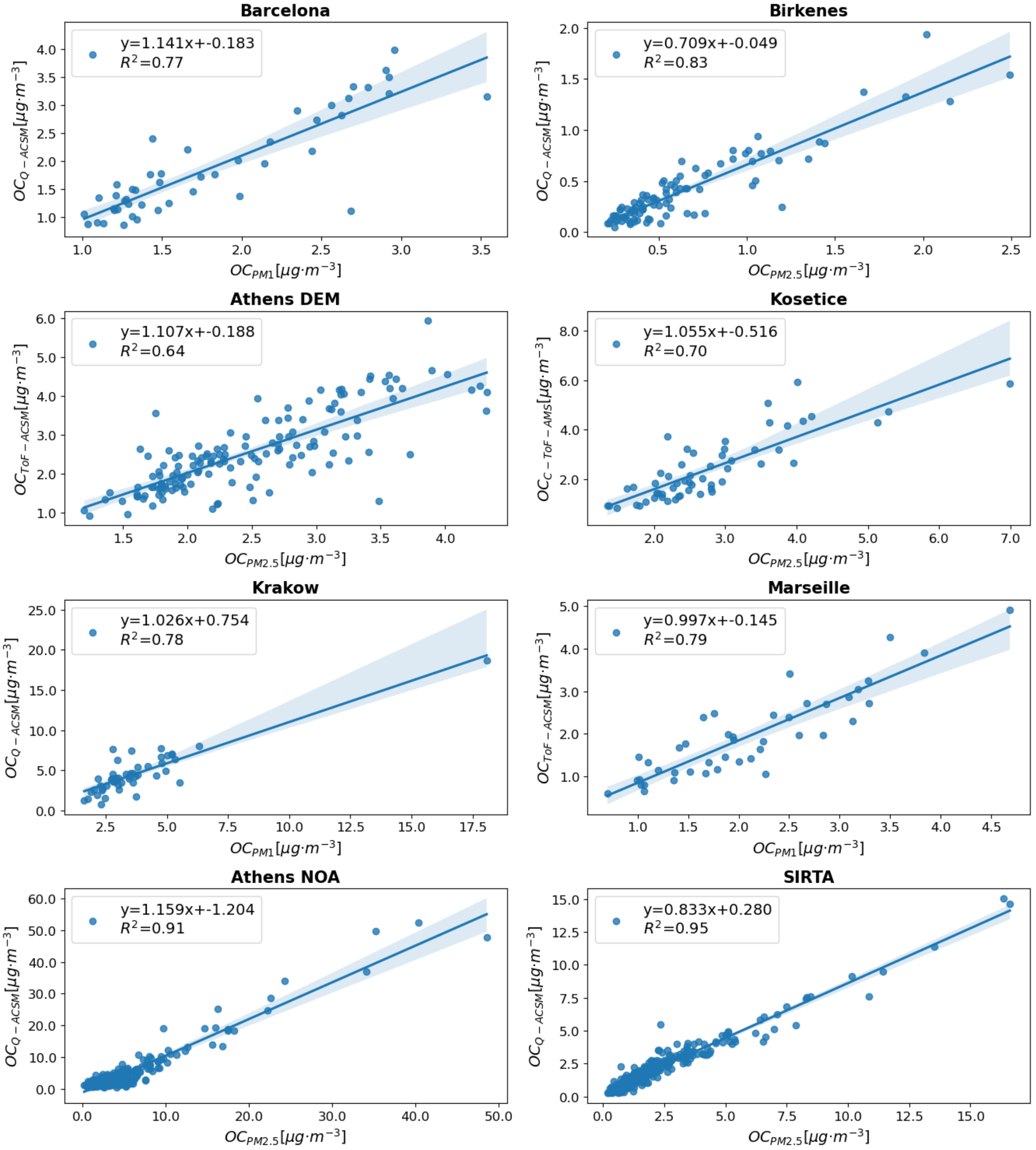

Figure S2: **Relationships between OC_{PM} and OC_{ACSM}.** Linear fits (slope, intercept and R^2) between OC_{PMx} (x=1 or 2.5 um) and OC_x (x=Q-ACSM, ToF-ACSM, C-ToF-AMS)

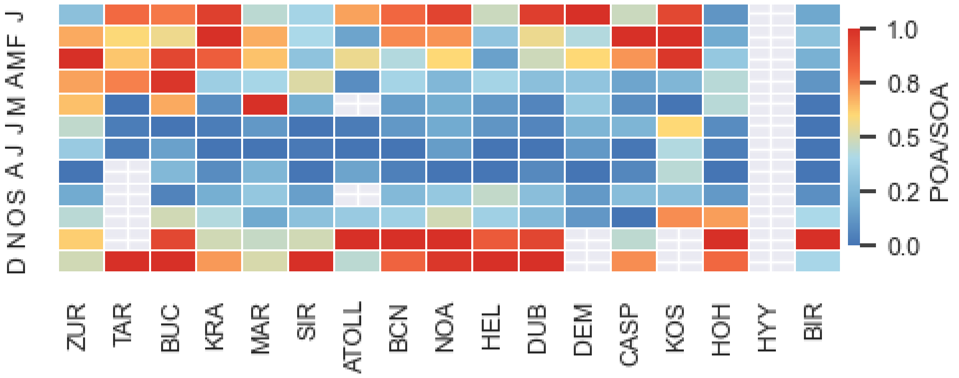

Figure S3: **Site-dependent normalized monthly POA/SOA ratios**. Normalized annual cycle of the POA/SOA ratio at the 17 measurement sites. Color code 0–1 indicates progressive increase from lowest value (blue) to highest value (red).

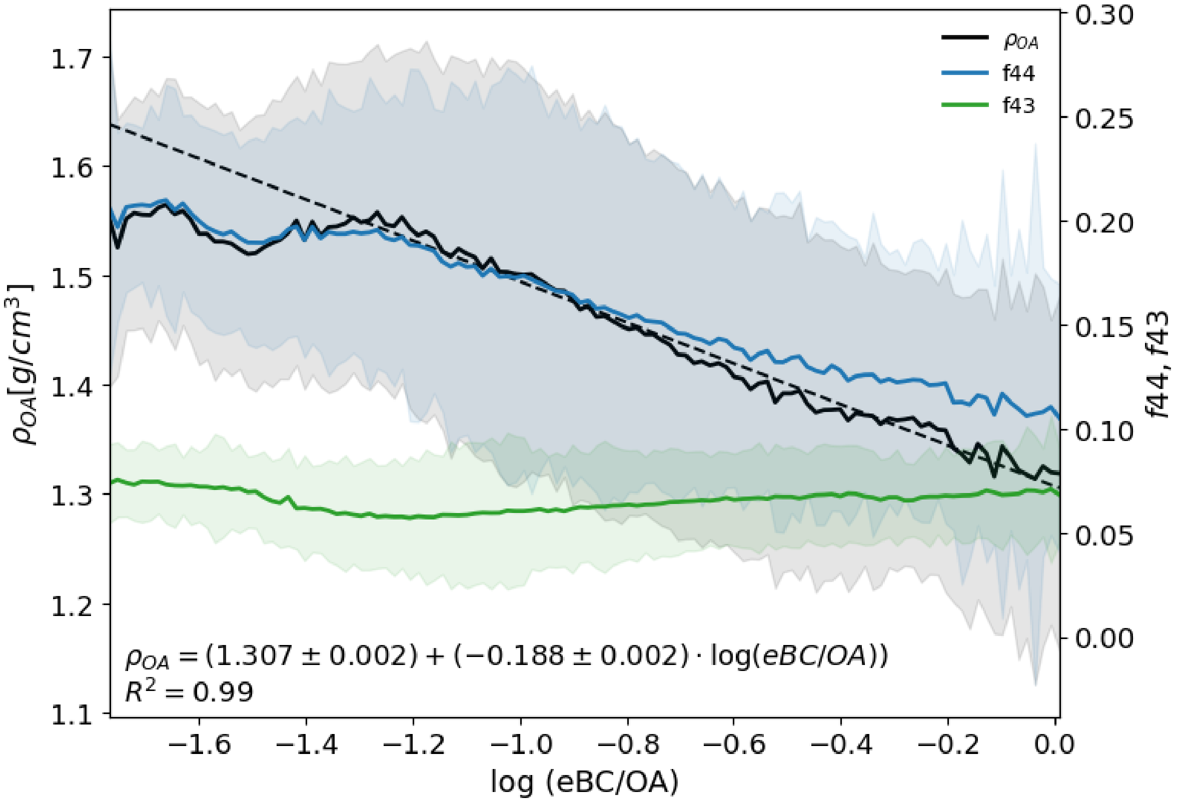

Figure S4: Parameterization of OA density and ACSM f44 and f43 as functions of the eBC/OA ratio. OA density ρ_{OA}, f43 and f44 as function of log(eBC/OA). Coloured areas represent the standard deviation of the data corresponding to each variable. Dashed black line is the linear fit of binned ρ_{OA} for log(eBC/OA) from -1.3 to 0.0.

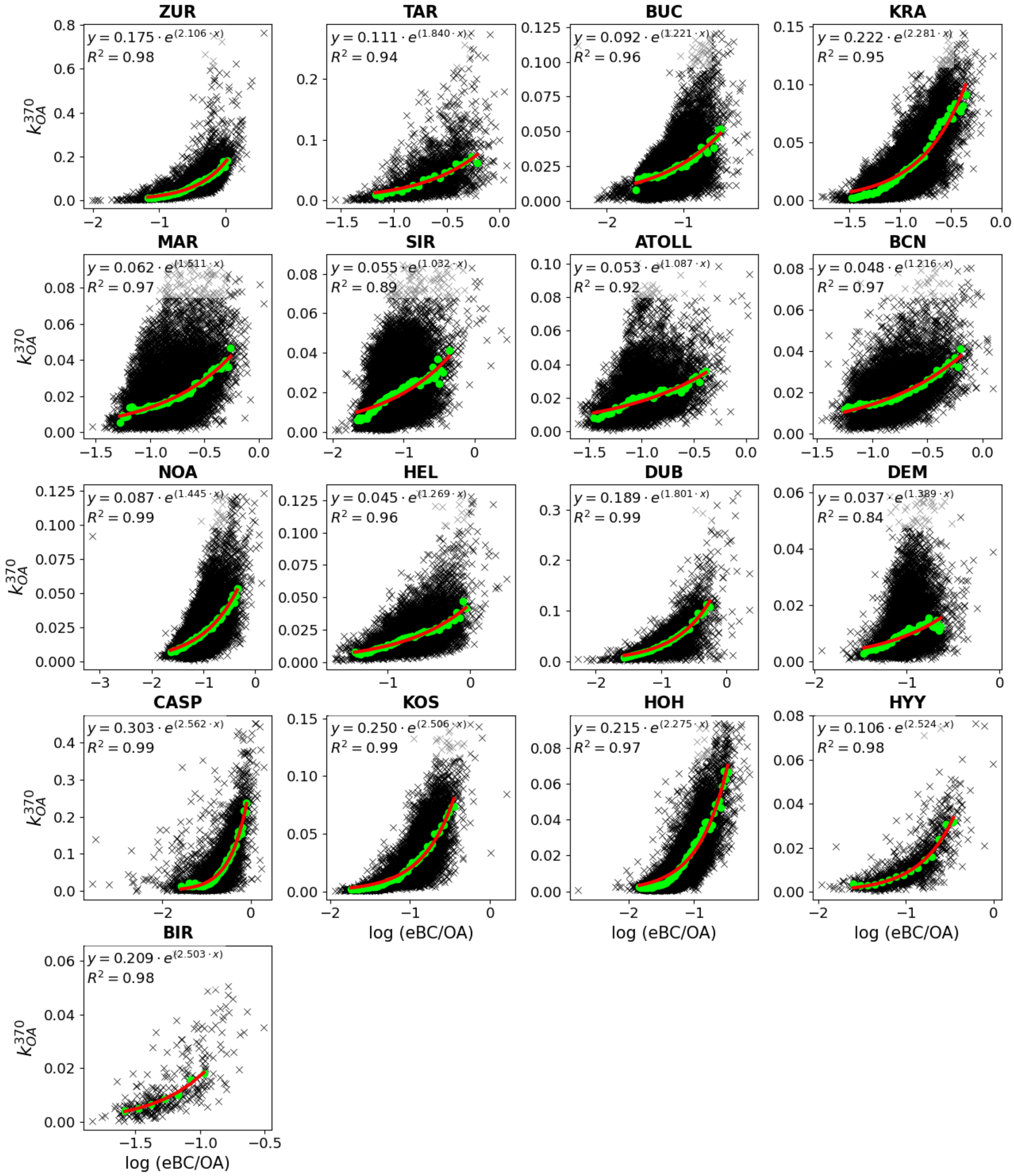

Figure S5: **SIte-dependent parameterizations of k_{OA} at 370 nm as a function of the eBC/OA ratio.** Dependence of k_{OA}^{370} on log(eBC/OA) at the measurement sites considered in this work. Black crosses are the hourly experimental data, green dots correspond to the binned k_{OA}^{370} and log(eBC/OA) values, and red lines represent the exponential fits. The parameters of the exponential fits are reported in each plot.

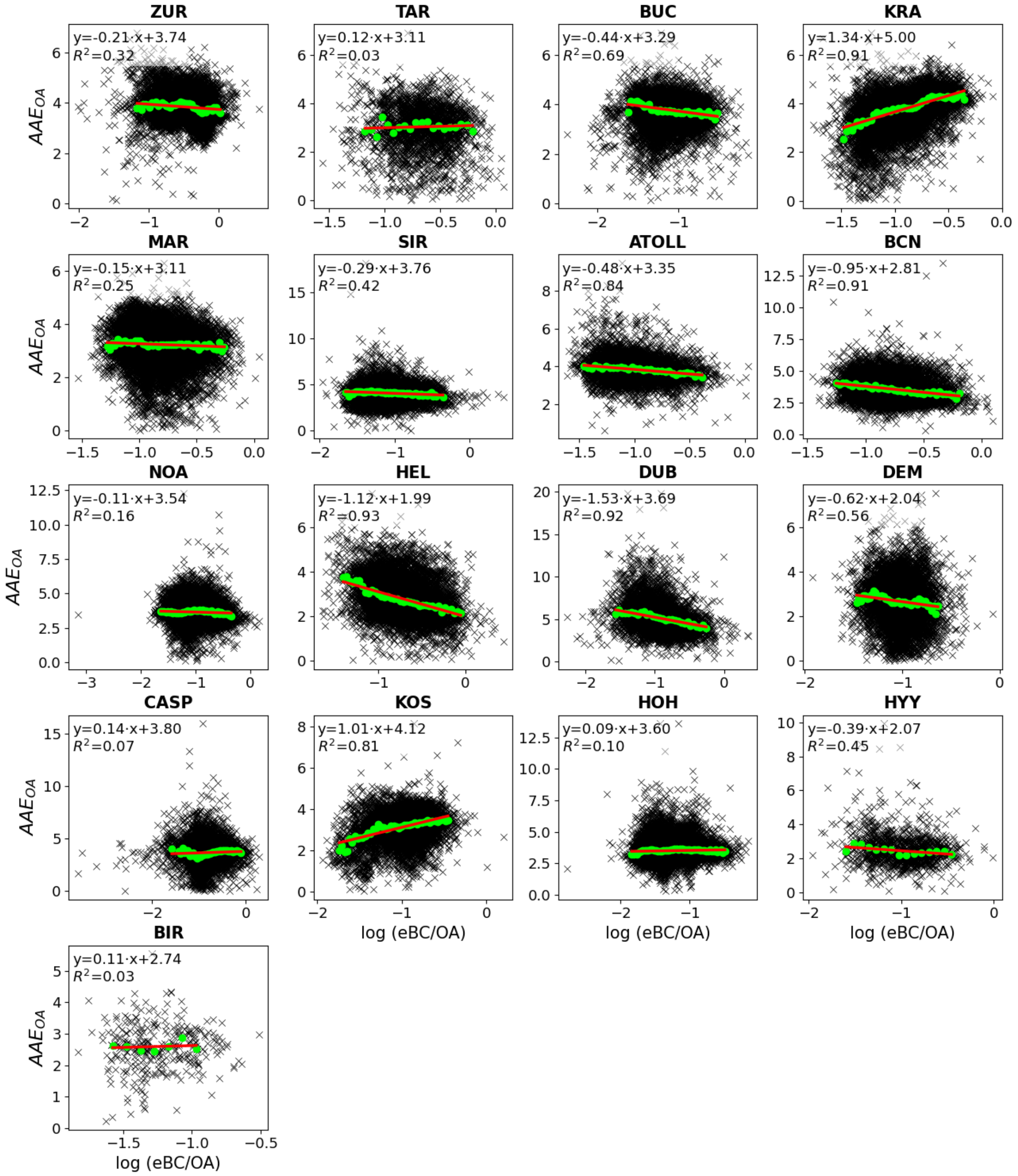

Figure S6: **Site-dependent parameterizations of AAE_{OA} as a function of the eBC/OA ratio.** Dependence of AAE_{OA} calculated between 370 nm and 590 nm on log(eBC/OA) at the measurement sites considered in this work. Black crosses are the hourly experimental data, green dots correspond to the binned AAE_{OA} and log(eBC/OA) values, and red lines represent the linear fits. The parameters of the linear fits are reported in each plot.

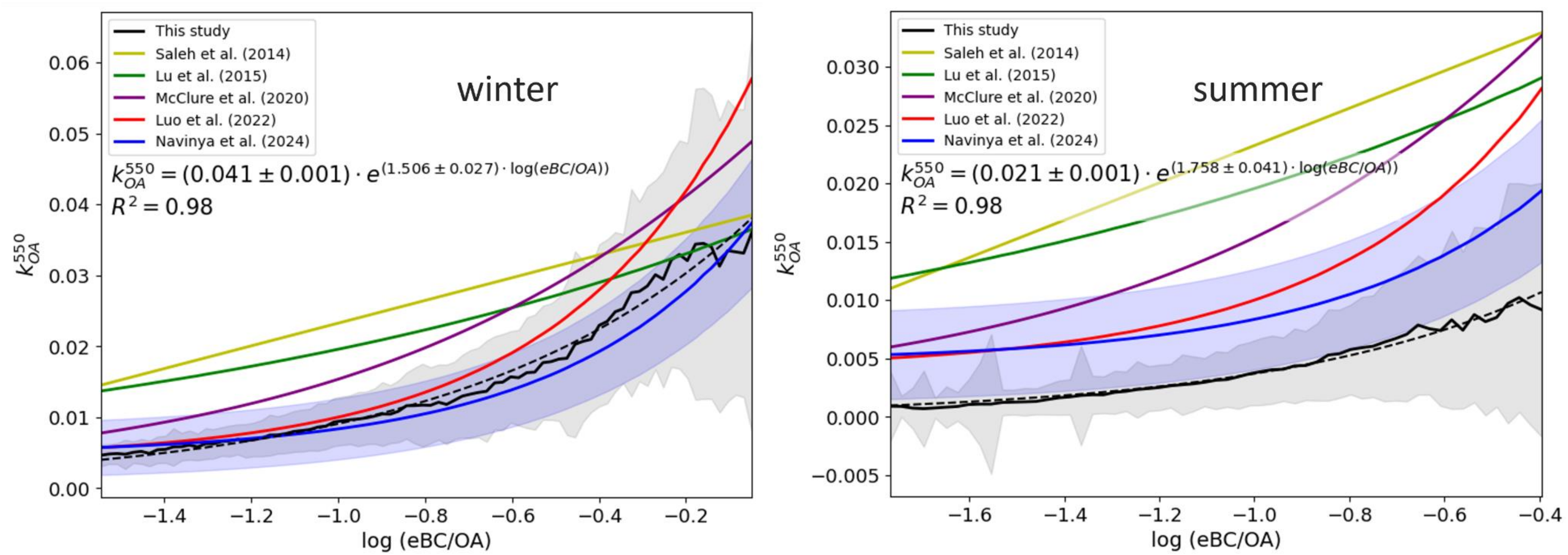

Figure S7: **Parameterization of k_{OA} at 550 nm as a function of the eBC/OA ratio for winter and summer.** Dependence of k_{OA}^{550} on log(eBC/OA) ratio for winter(left) and summer (right). Black line represents the binned k_{OA}^{550} obtained in this study using all available data and the dashed black line is the fit. Grey area is the standard deviation of the data in this study; red, dark green, light green, magenta and blue lines are the curves calculated using the parameterizations from Luo[1], Lu[2], Saleh[3], McClure[4], and Navinya[5], respectively. Blue area is the range of k_{OA}^{550} values based on the parameterization from Navinya[5].

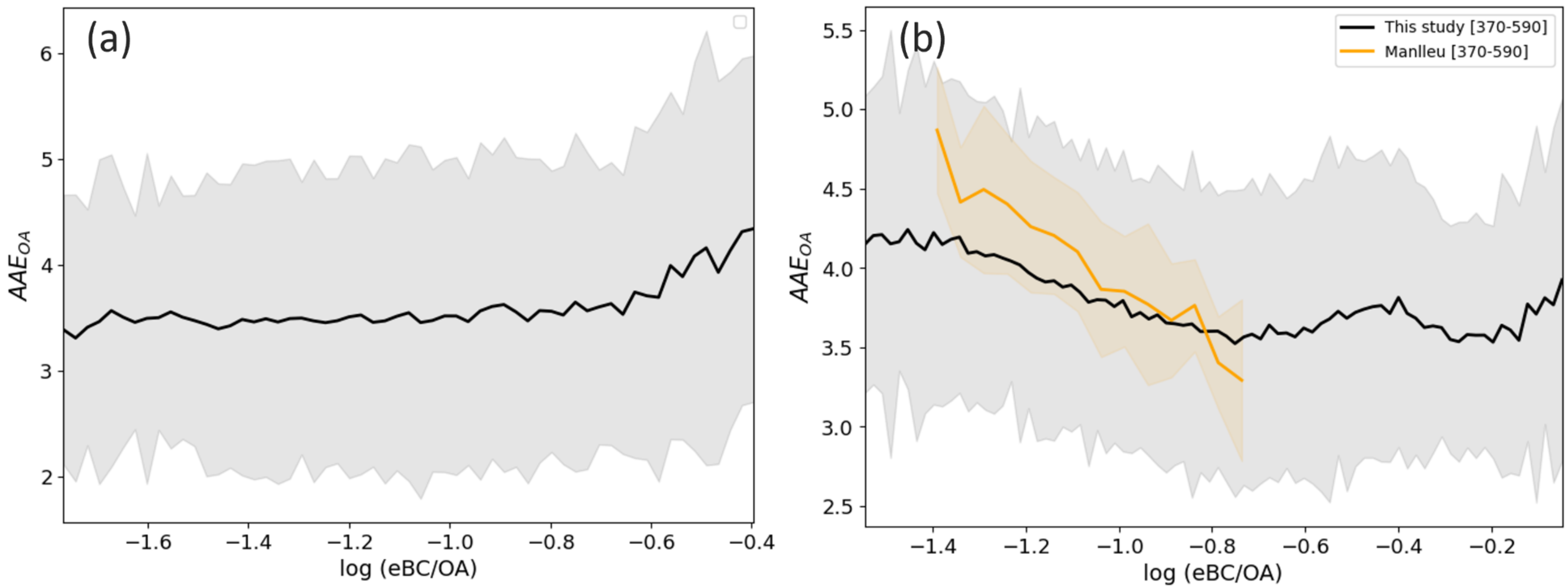

Figure S8: **Parameterization of AAE_{OA} as a function of the eBC/OA ratio for winter and summer.** (a) Dependence of AAE_{OA} (calculated between 370 nm and 590 nm) in summer on log(eBC/OA) using all available data (black line). (b) the same for winter (black line) and using data from Manlleu (orange line). Grey and orange areas represent the standard deviation using all available data and for Manlleu, respectively.

Table S1: Measurement site specifications: site name, acronym, latitude, longitude, altitude, measurement period, type, ACSM/AMS instrument.

Site	Acronym	Lat.	Lon.	Altitude	Period	Type	ACSM/AMS
Lille (FR)	ATOLL	50.61°N	3.14°E	230	2016-2017	urban	Q-ACSM
Barcelona (ES)	BCN	41.38°N	2.11°E	64	2017-2018	urban	Q-ACSM
Birkenes (NO)	BIR	58.39°N	8.25°E	219	2016-2018	non-urban	Q-ACSM
Bucharest (RO)	BUC	44.35°N	26.03°E	93	2017-2018	urban	Q-ACSM
Carnsore Point (IE)	CASP	52.17°N	6.36°W	9	2016-2017	non-urban	Q-ACSM
Demokritos (GR)	DEM	37.99°N	23.82°E	270	2017-2018	urban	ToF-ACSM
Dublin (IE)	DUB	53.31°N	6.22°W	20	2016-2017	urban	Q-ACSM
Helsinki (FI)	HEL	60.20°N	24.97°E	26	2017-2018	urban	Q-ACSM
Hohenpeissenberg (DE)	HOH	47.80°N	11.01°E	985	2017-2019	non-urban	Q-ACSM
Hyytiälä (FI)	HYY	61.85°N	24.29°E	181	2017-2018	non-urban	Q-ACSM
Kosetice (CZ)	KOS	49.58°N	15.08°E	534	2019	non-urban	C-ToF-AMS
Krakow (PL)	KRA	50.07°N	19.92°E	383	2018-2019	urban	Q-ACSM
Marseille (FR)	MAR	43.30°N	5.39°E	71	2017-2018	urban	ToF-ACSM
Athens-NOA (GR)	NOA	37.97°N	23.72°E	105	2016-2019	urban	Q-ACSM
SIRTA (FR)	SIR	48.70°N	2.15°E	162	2016-2017	urban	Q-ACSM
Tartu (EE)	TAR	58.37°N	26.73°E	70	2016-2017	urban	Q-ACSM
Zurich (CH)	ZUR	47.36°N	8.53°E	409	2016-2017	urban	Q-ACSM

Table S2: Mass absorption cross section (MAC), Absorption Angstrom Exponent (AAE) and imaginary refractive index (k) of bulk OA and MAC and AAE of OA sources. All reported MAC values of OA sources from MLR showed p<0.001; nd: OA source not detected from ACSM data; 0: OA source detected from ACSM data but excluded from multilinear regression analysis (i.e. MAC =0).

bulk OA	λ	DEM	NOA	BCN	MAR	HEL	TAR	ATOLL	ZUR	DUB	KRA	SIR	BUC	CASP	BIR	HOH	HYY	KOS
k	370	0.009	0.018	0.017	0.018	0.017	0.031	0.019	0.046	0.016	0.021	0.019	0.021	0.029	0.009	0.010	0.007	0.021
MAC	370	0.342	0.633	0.657	0.714	0.615	1.174	0.664	1.733	0.458	0.722	0.673	0.744	0.989	0.267	0.347	0.296	0.756
	470	0.228	0.418	0.340	0.390	0.434	0.638	0.311	0.778	0.268	0.384	0.244	0.326	0.482	0.171	0.154	0.216	0.489
	520	0.157	0.174	0.218	0.230	0.245	0.403	0.159	0.429	0.123	0.208	0.150	0.195	0.276	0.103	0.095	0.147	0.388
	590	0.110	0.118	0.126	0.165	0.166	0.295	0.118	0.275	0.093	0.127	0.102	0.133	0.165	0.079	0.068	0.105	0.190
AAE	370-590	2.64	3.64	3.64	3.24	2.91	3.01	3.86	4.01	5.20	3.78	4.13	3.76	3.66	2.56	3.56	2.42	3.25
**OA sources**	**λ**	**DEM**	**NOA**	**BCN**	**MAR**	**HEL**	**TAR**	**ATOLL**	**ZUR**	**DUB**	**KRA**	**SIR**	**BUC**	**CASP**	**BIR**	**HOH**	**HYY**	**KOS**
BBOA/ Wood (MAC)	370	1.761	3.334	2.886	4.474	*nd*	5.496	6.064	7.164	3.729	2.852	3.518	3.064	5.038	1.721	2.637	*nd*	6.713
	470	0.630	1.084	1.193	1.615	*nd*	1.910	2.298	2.977	1.906	1.406	0.960	1.014	2.420	1.191	1.150	*nd*	3.042
	520	0.368	0.491	0.716	0.980	*nd*	1.193	1.309	1.877	1.337	0.849	0.592	0.602	1.330	0.718	0.745	*nd*	1.717
	590	0.214	0.293	0.386	0.537	*nd*	0.866	0.808	1.207	1.010	0.493	0.312	0.376	0.930	0.569	0.536	*nd*	1.077
AAE	370-590	4.54	5.33	4.29	4.54		4.06	4.37	3.85	2.85	3.75	5.19	4.56	3.71	2.45	3.48		3.97
HOA (MAC)	370	0	0.427	1.209	2.122	1.032	2.036	0	1.101	2.540	1.169	0.889	0.262	4.231	0	1.336	*nd*	0.565
	470	0	0.443	0.970	1.280	1.045	0.811	0	0.503	1.543	1.039	0.739	0.193	1.878	0	0.544	*nd*	0.429
	520	0	0.156	0.565	0.707	0.534	0.323	0	0.161	0.732	0.384	0.502	0.099	1.016	0	0.319	*nd*	0.229
	590	0	0.113	0.305	0.523	0.371	0.128	0	0.060	0.250	0.205	0.402	0.072	0.669	0	0.251	*nd*	0.150
AAE	370-590		3.01	2.88	3.09	2.24	5.91		6.23	4.80	3.78	1.74	2.86	4.03		3.72		2.87

**CCOA (MAC)**	370	*nd*	*nd*	*nd*	*nd*	*nd*	*nd*	*nd*	*nd*	2.150	5.816	*nd*	*nd*	4.658	*nd*	*nd*	*nd*	*nd*
	470	*nd*	*nd*	*nd*	*nd*	*nd*	*nd*	*nd*	*nd*	0.834	2.273	*nd*	*nd*	2.213	*nd*	*nd*	*nd*	*nd*
	520	*nd*	*nd*	*nd*	*nd*	*nd*	*nd*	*nd*	*nd*	0.397	1.254	*nd*	*nd*	1.226	*nd*	*nd*	*nd*	*nd*
	590	*nd*	*nd*	*nd*	*nd*	*nd*	*nd*	*nd*	*nd*	0.145	0.805	*nd*	*nd*	0.857	*nd*	*nd*	*nd*	*nd*
**AAE**	370-590									5.68	4.31			3.72				
**COA (MAC)**	370	0	0.530	1.372	0	*nd*	*nd*	*nd*	0	*nd*	*nd*	*nd*	*nd*	*nd*	*nd*	*nd*	*nd*	*nd*
	470	0	0.094	0.434	0	*nd*	*nd*	*nd*	0	*nd*	*nd*	*nd*	*nd*	*nd*	*nd*	*nd*	*nd*	*nd*
	520	0	0.053	0.233	0	*nd*	*nd*	*nd*	0	*nd*	*nd*	*nd*	*nd*	*nd*	*nd*	*nd*	*nd*	*nd*
	590	0	0.026	0.114	0	*nd*	*nd*	*nd*	0	*nd*	*nd*	*nd*	*nd*	*nd*	*nd*	*nd*	*nd*	*nd*
**AAE**	370-590		6.50	5.32														
**MO-OOA (MAC)**	370	0.619	0.707	0.247	0.693	0.188	0	0	0	0.323	0	0.256	0.248	0	*nd*	0	0.479	0
	470	0.394	0.373	0.182	0.294	0.100	0	0	0	0.185	0	0.071	0.096	0	*nd*	0	0.334	0
	520	0.294	0.156	0.142	0.186	0.070	0	0	0	0.118	0	0.046	0.066	0	*nd*	0	0.214	0
	590	0.221	0.110	0.098	0.147	0.057	0	0	0	0.088	0	0.039	0.052	0	*nd*	0	0.155	0
**AAE**	370-590	2.22	4.15	1.93	3.44	2.64				2.84		4.20	3.46				2.45	
**LO-OOA (MAC)**	370	0	0	0.803	0	1.709	0	0.553	1.933	0.294	0	0.574	1.639	0	*nd*	0	0	0
	470	0	0	0.668	0	0.959	0	0.362	0.564	0.160	0	0.223	0.706	0	*nd*	0	0	0
	520	0	0	0.464	0	0.579	0	0.216	0.332	0.071	0	0.126	0.432	0	*nd*	0	0	0

	590	0	0	0.286	0	0.378	0	0.172	0.200	0.022	0	0.089	0.296	0	*nd*	0	0	0
**AAE**	370-590			2.14		3.26		2.59	4.92	5.41		4.11	3.73					
**Peat (MAC)**	370	*nd*	*nd*	*nd*	*nd*	*nd*	*nd*	*nd*	*nd*	2.361	*nd*	*nd*	*nd*	2.574	*nd*	*nd*	*nd*	*nd*
	470	*nd*	*nd*	*nd*	*nd*	*nd*	*nd*	*nd*	*nd*	0.630	*nd*	*nd*	*nd*	1.166	*nd*	*nd*	*nd*	*nd*
	520	*nd*	*nd*	*nd*	*nd*	*nd*	*nd*	*nd*	*nd*	0.278	*nd*	*nd*	*nd*	0.631	*nd*	*nd*	*nd*	*nd*
	590	*nd*	*nd*	*nd*	*nd*	*nd*	*nd*	*nd*	*nd*	0.139	*nd*	*nd*	*nd*	0.408	*nd*	*nd*	*nd*	*nd*
**AAE**	370-590									6.15				4.02				

Table S3: Literature MAC_{OA} or MAE_{OA}, k_{OA} and ρ_{OA} values used for Figure 2. (*) 365 nm; (**) 370 nm; (***) 375 nm

Reference	Experiment	MAC_{OA} | MAE_{OA} (m^2/g)	k_{OA}	ρ_{OA} (g/cm^3)
Atwi et al. (2022)[6]	Chamber experiment			1.2
Chakrabarty et al. (2023)[7]	Chamber experiment		0.116 (**)	1.4
				1.6
Chen et al. (2020)[8]	Ambient	0.75 (*)		
		1.12 (*)		
Cheng et al. (2016)[9]	Ambient	1.22 (*)		
		1.45 (*)		
Cheng et al. (2017)[10]	Ambient	0.44 (*)		1.2
		0.58 (*)		
Cheng et al. (2019)[11]	Chamber experiment			1.2
				1.3
				1.8
Choudhary et al. (2017)[12]	Ambient	1.5 (*)		1.65
Choudhary et al. (2018)[13]	Ambient	1.6 (*)		1.65
		1.8 (*)		
Choudhary et al. (2021)[14]	Ambient			1.2
Corbin et al. (2019)[15]	Chamber experiment			1.8
Cuesta-Mosquera et al. (2024)[16]	Ambient	2.4 (**)		1.4
Debbarma et al. (2024)[17]	Ambient	0.3 (**)		
		1.2 (**)		
Deng et al. (2022)[18]	Ambient		0.076 (*)	1.5
			0.041 (*)	
Devaprasad et al. (2024)[19]	Ambient		0.041 (*)	1.5
			0.035 (*)	
Feng et al. (2013)[20]	Chamber experiment			1.568
Fröhlich et al. (2015)[21]	Ambient			1.3
He et al. (2022)[22]	Chamber experiment			1.35
Islam et al. (2022)[23]	Near sources	0.48 (*)		1.2
Jiang et al. (2022)[24]	Ambient	0.53 (***)		
		0.56 (***)		
Kasthuriarachchi et al. (2020)[25]	Ambient	0.5 (**)		
		0.67 (**)		
		0.85 (**)		
		0.97 (**)		
		1.13 (**)		
		1.66 (**)		
Kirillova et al. (2016)[26]	Ambient	0.52 (*)		
		0.57 (*)		
		0.59 (*)		
		0.68 (*)		
Kumar et al. (2018)[27]	Chamber experiment	2.2 (**)		1.2
		5.5 (**)		1.3
				1.6
				1.5
				1.38
				1.8

Lei et al. (2018)[28]	Ambient	1.4 (*)		
Li et al. (2016)[29]	Chamber experiment			1.2
Liakakou et al. (2020)[30]	Ambient	3.77 (**)		
Lu et al. (2015)[31]	Chamber experiment			1.2
Luo et al. (2022)[32]	Near sources	2.46 (**)	0.13 (**)	1.25
Moschos et al. (2021)[33]	Ambient			1.5
Nakao et al. (2013)[34]	Chamber experiment			1.48
				1.8
				1.22
Navinya et al. (2024)[35]	Near sources	2.362 (*)	0.1029 (*)	1.5
		2.087 (*)	0.0906 (*)	
		2.060 (*)	0.0898 (*)	
		1.333 (*)	0.0584 (*)	
Ponczek et al. (2022)[36]	Ambient			1.8
Poulain et al. (2020)[37]	Ambient			1.77
Rana et al. (2020)[38]	Near sources			1.5
Runa et al. (2021)[39]	Chamber experiment			1.65
Saleh et al. (2013)[40]	Chamber experiment			1.8
Saleh et al. (2015)[41]	Chamber experiment			1.2
Sand et al. (2021)[42]	Chamber experiment			1.8
Sarkar et al. (2019)[43]	Ambient	1.2 (*)		
Shamjad et al (2016)[44]	Ambient			1.5
Shen et al. (2024)[45]	Near sources			1.4
				1.7
Tian et al. (2023)[46]	Ambient	1.43 (**)		
		2.78 (**)		
Wu et al. (2020)[47]	Ambient	1.2 (*)	0.048 (*)	1.5
		1.2 (*)	0.038 (*)	
		1.3 (*)	0.033 (*)	
		1.3 (*)	0.021 (*)	
Xie et al. (2018)[48]	Chamber experiment	4.2 (*)		
		5.09 (*)		
Zeng et al. (2022)[49]	Ambient			1.4
				1.8
Zhang et al. (2018)[50]	Ambient			1.8
Zhang et al. (2022)[51]	Ambient			1.8

References

1. Luo, B. *et al.* Parameterizations of size distribution and refractive index of biomass burning organic aerosol with black carbon content. *Atmos. Chem. Phys.* **22**, 12401–12415 (2022).

2. Lu, Z. *et al.* Light absorption properties and radiative effects of primary organic aerosol emissions. *Environ. Sci. Technol.* **49**, 4868–4877 (2015).

3. Saleh, R. *et al.* Brownness of organics in aerosols from biomass burning linked to their black carbon content. *Nat. Geosci.* **7**, 647–650 (2014).

4. McClure, C. D. *et al.* Biomass-burning-derived particles from a wide variety of fuels – Part 1: Properties of primary particles. *Atmos. Chem. Phys.* **20**, 1531–1547 (2020).

5. Navinya, C. *et al.* Brownness of organics in anthropogenic biomass burning aerosols over South Asia. *Atmos. Chem. Phys.* **24**, 13285–13297 (2024).

6. Atwi, K., Cheng, Z., El Hajj, O., Perrie, C. & Saleh, R. A dominant contribution to light absorption by methanol-insoluble brown carbon produced in the combustion of biomass fuels typically consumed in wildland fires in the United States. *Environ. Sci.: Atmos.* **2**, 182–191 (2022).

7. Chakrabarty, R. K. *et al.* Shortwave absorption by wildfire smoke dominated by dark brown carbon. *Nat. Geosci.* **16**, 683–688 (2023).

8. Chen, Y. *et al.* Brown carbon in atmospheric fine particles in Yangzhou, China. *Atmos. Res.* **244**, 105028 (2020).

9. Cheng, Y. *et al.* The characteristics of brown carbon aerosol during winter in Beijing. *Atmos. Environ.* **127**, 355–364 (2016).

10. Cheng, Y. et al. Brown and black carbon in Beijing aerosol. *Sci. Total Environ.* **599–600**, 1047–1055 (2017).

11. Cheng, Z., Atwi, K., Onyima, T. & Saleh, R. Investigating the dependence of light-absorption properties of combustion carbonaceous aerosols on combustion conditions. *Aerosol Sci. Technol.* **53**, 419–434 (2019).

12. Choudhary, V., Rajput, P., Rajeev, P., & Gupta, T. Synergistic effect in absorption properties of brown carbon and elemental carbon over IGP during weak south-west monsoon. *Aerosol Sci. Engin.* **1**, 138–149 (2017).

13. Choudhary, V., Rajput, P., Singh, D.K., Singh, A.K. & Gupta, T. Light absorption characteristics of brown carbon during foggy and non-foggy episodes over the Indo-Gangetic Plain. *Atmos. Pollut. Res.* **9**, 494–501 (2018).

14. Choudhary, V., Singh, G.K., Gupta, T., & Paul, D. Absorption and radiative characteristics of brown carbon aerosols during crop residue burning in the source region of Indo-Gangetic Plain. *Atmos. Res.* **249**, 105285 (2021).

15. Corbin, J.C. *et al.* Infrared-absorbing carbonaceous tar can dominate light absorption by marine-engine exhaust. *npj Clim. Atmos. Sci.* **2**, 1–8 (2019).

16. Cuesta-Mosquera, A. *et al.* Optical properties and simple forcing efficiency of the organic aerosols and black carbon emitted by residential wood burning in rural central Europe. *Atmos. Chem. Phys.* **24**, 2583–2605 (2024).

17. Debbarma, S., Raparthi, N., Venkataraman, C. & Phuleria, H.C., Characterization and apportionment of carbonaceous aerosol emission factors from light-duty and heavy-duty vehicle fleets in Maharashtra, India. *Environ. Pollut.* **345**, 123479 (2024).

18. Deng, J. *et al.* Measurement report: Optical properties and sources of water-soluble brown carbon in Tianjin, North China – insights from organic molecular compositions. *Atmos. Chem. Phys.* **22**, 6449–6470 (2022).

19. Devaprasad, M. *et al.* Dual carbon isotope-based brown carbon aerosol characteristics at a high-altitude site in the northeastern Himalayas: Role of biomass burning. *Sci. Total Environ.* **912**, 169451 (2024).

20. Feng, Y., Ramanathan, V. & Kotamarthi, V. R. Brown carbon: a significant atmospheric absorber of solar radiation? *Atmos. Chem. Phys.* **13**, 8607–8621 (2013).

21. Fröhlich, R. *et al.* The ToF-ACSM: a portable aerosol chemical speciation monitor with TOFMS detection. *Atmos. Meas. Tech.* **6**, 3225–3241 (2013).

22. He, Q. *et al.* Optical Properties of Secondary Organic Aerosol Produced by Photooxidation of Naphthalene under NOx Condition. *Environ. Sci. Tech.* **56**, 4816–4827 (2022).

23. Islam, M.M., Neyestani, S.E., Saleh, R. & Grieshop, A.P. Quantifying brown carbon light absorption in real-world biofuel combustion emissions. *Aerosol Sci. Technol.* **56**, 502–516 (2022).

24. Jiang, X. *et al.* Connecting the Light Absorption of Atmospheric Organic Aerosols with Oxidation State and Polarity.*Environ. Sci. Tech.* **56**, 12873–12885 (2022).

25. Kasthuriarachchi, N., Rivellini, L.-H., Adam, M.G. & Lee, A.K.Y. Light absorbing properties of primary and secondary brown carbon in a tropical urban environment. *Environ. Sci. Technol.* **54**, 10808–10819 (2020).

26. Kirillova, E.N. *et al.* Light absorption properties of brown carbon in the high Himalayas. *J. Geophys. Res. Atmos.* **121**, 9621–9639 (2016).

27. Kumar, N.K. *et al.* Production of particulate brown carbon during atmospheric aging of residential wood-burning emissions. *Atmos. Chem. Phys.* **18**, 17843–17861 (2018).

28. Lei, Y. *et al.* Optical characteristics and source apportionment of brown carbon in winter PM2.5 over Yulin in Northern China. *Atmos. Res.* **213**, 27–33 (2018).

29. Li, X., Chen, Y. & Bond, T.C. Light absorption of organic aerosol from pyrolysis of corn stalk. *Atmos. Environ.* **144**, 249–256 (2016).

30. Liakakou, E. *et al.* Long-term variability, source apportionment and spectral properties of black carbon at an urban background site in Athens, Greece. *Atmos. Environ.* **222**, 117137 (2020).

31. Lu, Z. *et al.* Light absorption properties and radiative effects of primary organic aerosol emissions. *Environ. Sci. Technol.* **49**, 4868–4877 (2015).

32. Luo, B. *et al.* Parameterizations of size distribution and refractive index of biomass burning organic aerosol with black carbon content. *Atmos. Chem. Phys.* **22**, 12401–12415 (2022).

33. Moschos, V. *et al.* Source-specific light absorption by carbonaceous components in the complex aerosol matrix from yearly filter-based measurements. *Atmos. Chem. Phys.* **21**, 12809–12833 (2021).

34. Nakao, S. *et al.* Density and elemental ratios of secondary organic aerosol: Application of a density prediction method. *Atmos. Environ.* **68**, 273–277 (2012).

35. Navinya, C. *et al.* Brownness of organics in anthropogenic biomass burning aerosols over South Asia. *Atmos. Chem. Phys.* **24**, 13285–13297 (2024).

36. Ponczek, M. *et al.* Linking the chemical composition and optical properties of biomass burning aerosols in Amazonia. *Environ. Sci. Atmos.* **2**, 252–269 (2022).

37. Poulain, L. *et al.* Multi-year ACSM measurements at the central European research station Melpitz (Germany) – Part 1: Instrument robustness, quality assurance, and impact of upper size cutoff diameter. *Atmos. Meas. Tech.* **13**, 4973–4994 (2020).

38. Rana, A. *et al*. Optical properties of aerosol brown carbon (BrC) in the eastern Indo-Gangetic Plain. *Sci. Total Environ*. **716**, 137102–137102 (2020).

39. Runa, F., Islam, Md.S., Jeba, F. & Salam, A. Light absorption properties of brown carbon from biomass burning emissions. *Environ. Sci. Pollut. Res.* **29**, 21012-21022 (2021).

40. Saleh, R. *et al.* Absorptivity of brown carbon in fresh and photo-chemically aged biomass-burning emissions. *Atmos. Chem. Phys.* **13**, 7683–7693 (2013).

41. Saleh, R. *et al.* Contribution of brown carbon and lensing to the direct radiative effect of carbonaceous aerosols from biomass and biofuel burning emissions. *J. Geophys. Res. Atmos.* **120**, 10285-10296 (2015).

42. Sand, M. *et al.* Aerosol absorption in global models from AeroCom phase III. *Atmos. Chem. Phys.* **21**, 15929–15947 (2021).

43. Sarkar, C., Venkataraman, C., Yadav, S., Phuleria, H.C. & Chatterjee, A. Origin and properties of soluble brown carbon in freshly emitted and aged ambient aerosols over an urban site in India. *Environ. Pollut.* **254**, 113077 (2019).

44. Shamjad, P.M., Tripathi, S.N., Thamban, N.M. & Vreeland, H. Refractive Index and Absorption Attribution of Highly Absorbing Brown Carbon Aerosols from an Urban Indian City-Kanpur. *Sci. Rep.* **6**. 37735 (2016).

45. Shen, Y. *et al.* Understanding the mechanism and importance of brown carbon bleaching across the visible spectrum in biomass burning plumes from the WE-CAN campaign. *Atmos. Chem. Phys.* **24**, 12881–12901 (2024).

46. Tian, J. *et al.* Impacts of biomass burning and photochemical processing on the light absorption of brown carbon in the southeastern Tibetan Plateau. *Atmos. Chem. Phys.* **23**, 1879–1892 (2023).

47. Wu, C. *et al.* The characteristics of atmospheric brown carbon in Xi'an, inland China: sources, size distributions and optical properties. *Atmos. Chem. Phys.* **20**, 2017–2030 (2020).

48. Xie, M., Shen, G., Holder, A.L., Hays, M.D. & Jetter, J.J. Light absorption of organic carbon emitted from burning wood, charcoal, and kerosene in household cookstoves. *Environ. Pollut.* **240**, 60–67 (2018).

49. Zeng, L. *et al.* Characteristics and evolution of brown carbon in western United States wildfires. *Atmos. Chem. Phys.* **22**, 8009–8036 (2022).

50. Zhang, Y. *et al.* Evidence of major secondary organic aerosol contribution to lensing effect black carbon absorption enhancement. *npj Clim Atmos Sci* **1**, 47 (2018)

51. Zhang, L. *et al.* Light absorption by brown carbon over the South-East Atlantic Ocean. *Atmos. Chem. Phys.* **22**, 9199–9213 (2022).
